# Transcriptional regulator MarT negatively regulates MarT-regulated motility gene I, a new gene involved in invasion and virulence of *Salmonella enterica*

**DOI:** 10.3389/fmicb.2024.1430982

**Published:** 2024-08-15

**Authors:** Sebastián A. Jerez, Aracely Y. Mora, Ana R. Millanao, Claudia P. Saavedra, Sergio A. Bucarey, Guido C. Mora, Nicolás A. Villagra, Alejandro A. Hidalgo

**Affiliations:** ^1^Programa de Doctorado en Biociencias Moleculares, Universidad Andres Bello, Santiago, Chile; ^2^Advanced Center for Chronic Diseases, Facultad de Ciencias Químicas y Farmacéuticas & Facultad de Medicina, Universidad de Chile, Santiago, Chile; ^3^Instituto de Farmacia, Facultad de Ciencias, Universidad Austral de Chile, Valdivia, Chile; ^4^Laboratorio de Microbiología Molecular, Facultad de Ciencias de la Vida, Universidad Andres Bello, Santiago, Chile; ^5^Departamento de Ciencias Biológicas Animales, Facultad de Ciencias Veterinarias y Pecuarias, Universidad de Chile, Santiago, Chile; ^6^Instituto de Investigación Interdisciplinar en Ciencias Biomédicas SEK (I3CBSEK), Facultad de Ciencias de la Salud, Universidad SEK, Santiago, Chile; ^7^Escuela de Tecnología Médica, Facultad de Salud, Universidad Santo Tomas, Santiago, Chile; ^8^Escuela de Química y Farmacia, Facultad de Medicina, Universidad Andres Bello, Santiago, Chile

**Keywords:** *Salmonella typhi*, *Salmonella typhimurium*, *marT*, pseudogene, MCP-family chemosensor

## Abstract

The speciation of *Salmonella* occurred by acquisition of genomic islands from other bacterial species and continued to diverge into subspecies and serovars with diferent range of host. *S. enterica* serovar Typhimurium (STM) is a generalist pathogen infecting hosts that include birds, mice, and humans, whilst *S. enterica* serovar Typhi (STY) is a restricted-host pathogen, infecting only humans. Despite their ranges of hosts, STM and STY possess 97–98% identity. Gain of genes by horizontal transference and loss of genes by mutations, are believed essential for differentiation of *Salmonella*. *Salmonella* pathogenicity island 3 (SPI-3) is an example combining these two processes. SPI-3 encodes *misL* and *marT*, among other genes. In STM, *misL* is required for gut colonization. Furthermore, protein MarT, positively regulates expression of misL by binding to *misL*-promoter. On the other hand, in SPI-3 of STY, *marT* and *misL* are pseudogenes. Interestingly, the gene t3766 (gene involved in resistance to H_2_O_2_) is present only in STY and is negatively regulated when *marT*_STM_ is heterologously expressed in STY. Based on the view that MarT might regulate genes implicated in virulence, this work searched for new genes regulated by MarT. *In silico* searches for possible MarT target genes were performed, and 4 genes were selected for further analysis as they contained at least 2 copies of the consensus MarT-binding sequence in their promoters. Mutating *marT* in STM or heterologously expressing *marT*_STM_ in STY confirmed that MarT negatively regulates ORF STY1408 or STM14_2003, its homologue in STM. STY1408 encodes for a putative protein with homology to methyl accepting chemotaxis proteins, which participate in chemotaxis and motility. Therefore, STY1408 was named *mrmI* (MarT-regulated motility gene I). Motility assays confirmed that the product of *mrmI* modulates motility. In addition, *in vitro* infection of cells with STM and STY mutants in *mrmI* reduces association with cells at 1, 3 and 24 h post-infection. Oral infection of mice showed that a *mrmI* null mutant was defective in producing systemic disease. Therefore, we conclude that MarT regulated *mrmI*, is involved in virulence of *Salmonella*. While pseudogenization of *marT* might modulate the fitness of narrow host range STY.

## 1 Introduction

**Figure 1:**
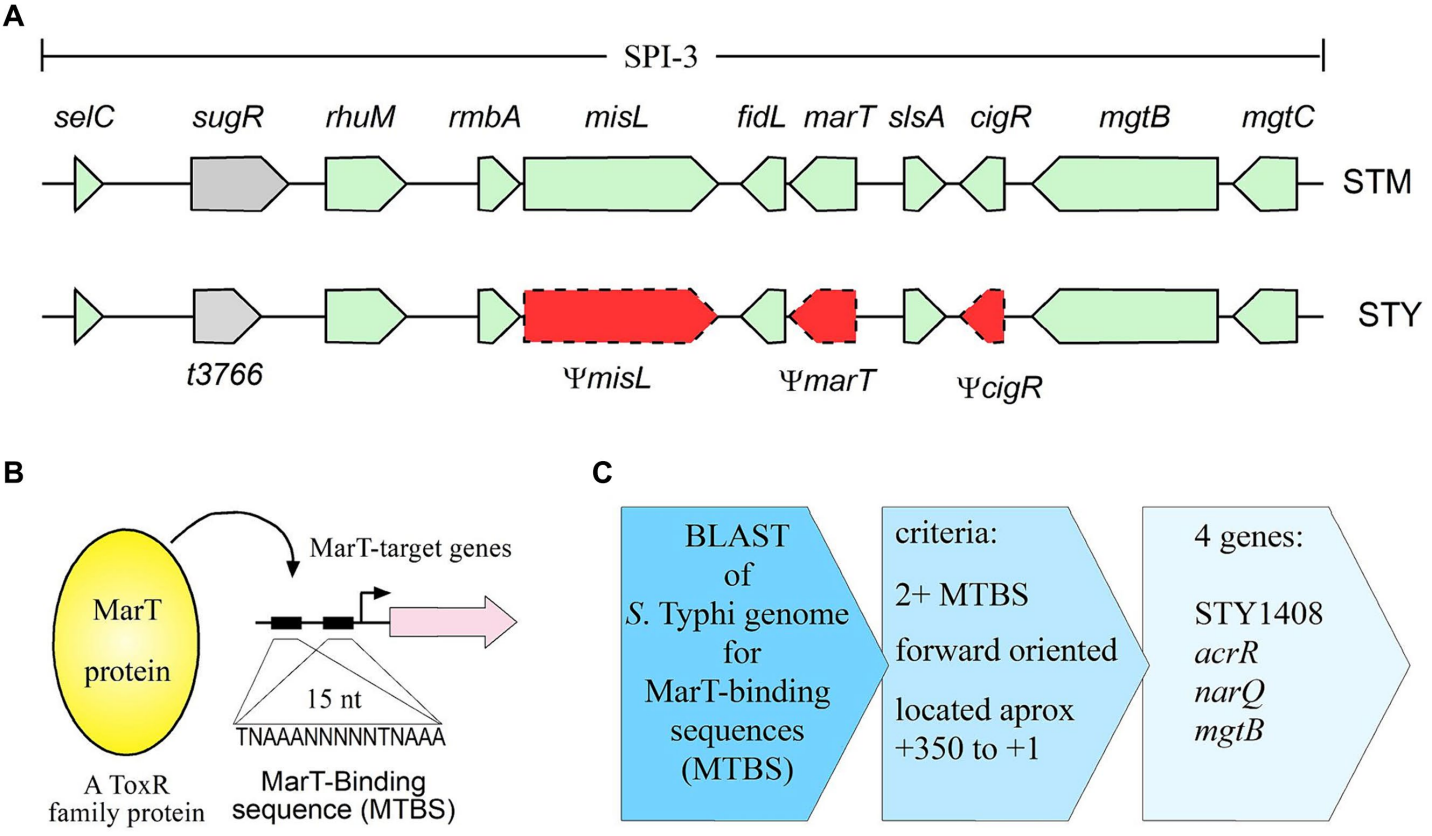
Genetic context of SPI-3 of *S. typhimurium* and *S. typhi* and flow chart depicting the search for MarT-binding sites in the vicinity of putative target genes. **(A)** Along with *misL* and *cigR*, *marT* is a pseudogene in *S. typhi* (STY), but not in *S. typhimurium* (STM). Gray shaded and green box, functional genes, red box with dotted lines, pseudogenes. **(B)** MarT is a ToxR-family transcription factor that binds to regulatory regions (MTBS) of target genes. **(C)** Schematic representation of the steps followed to identify genes regulated by MarT.

*Salmonella* is a genus consisting of only two species: *Salmonella bongori* which infects cold-blooded animals and *Salmonella enterica* which infects warm-blooded animals. The latter is divided into six subspecies, including the subspecies *enterica*, which includes serovars Typhimurium (*S. typhimurium* or STM, within the text) and Typhi (*S. typhi* or STY, within the text) (Boyd et al., 1993). STM is considered a generalist pathogen since it can infect birds, mice, and humans, among other hosts. In humans, STM produces self-limiting gastroenteritis, while in mice, it produces a systemic disease, resembling the fever symptoms of human typhoid. On the other hand, STY is a restricted host-range pathogen, because it only infects humans, producing the systemic disease typhoid fever which can be lethal if not treated. Despite the differences in host range and pathogenicity between both serovars, they possess at least 97% identity between their shared genes (McClelland et al., 2001; Parkhill et al., 2001). The limited differences between them could be explained by acquisition of new genes by Horizontal Gene Transfer (HGT) and the loss of genes by mutations. An example of these two genomic events is *Salmonella* Pathogenicity Island 3 (SPI-3), shown in Figure 1A. SPI-3 is a horizontally-acquired fragment of 17 kb localized at the *selC* locus of the chromosome, containing 10 functional genes in STM (Blanc-Potard et al., 1999). SPI-3 is also present in STY; however, in this serovar 3 of the 10 genes within SPI-3_STM_ are pseudogenes (Ψ*misL*, Ψ*marT* and Ψ*cigR*), and thus their full-length gene products are lost in STY. Furthermore, the gene t3766 (also named *surV*), involved in resistance to H_2_O_2_ is present only in STY SPI-3 (Tükel et al., 2007; Retamal et al., 2010; Ortega et al., 2016), in a clear example of combining gain and loss of genes, at only one genetic locus.

The *marT* gene (intact in STM, but inactivated in STY) encodes for a transcriptional regulator possessing 41% similarity with ToxR, a transcription factor of *Vibrio cholerae* which regulates the expression of cholera toxin, colonization factors and porins (Blanc-Potard et al., 1999). Transcription factors in the ToxR-family recognize the consensus sequence TNAAANNNNNTNAAA in the promoter of target genes (Goss et al., 2013). MarT positively regulates *misL* expression in STM by directly-binding to the sequence TNAAANNNNNTNAAA (Tükel et al., 2007). The *misL* gene, located in SPI-3, encodes for an adhesin required for the efficient colonization of birds and mice (Morgan et al., 2004; Dorsey et al., 2005). These observations suggest that MarT regulates the expression of genes involved in STM virulence. Transcriptomic analyses of STM showed that *marT*_STM_ expression is 10-times higher in bacteria harbored in macrophages (Eriksson et al., 2003) reinforcing the idea that *marT* is involved in the virulence of STM.

*In silico* analyses indicate that MarT is a protein of 260 amino acids, with a conserved DNA-binding domain between residues 32 and 180 (Tükel et al., 2007). However, *marT*_STY_ presents 12 nucleotide substitutions compared with *marT*_STM_ resulting in nonsense mutations and a truncated MarT_STY_ protein. The 60 nt truncated MarT_STY_ partially loses the DNA binding domain and the regulatory functions (Tükel et al., 2007; Ortega et al., 2016). Heterologous expression of *marT* downregulates *surV*, a gene located only in SPI-3 of STY that is involved in resistance to hydrogen peroxide (Ortega et al., 2016), again suggesting that *marT* is involved in virulence, by modulating transcription of target effector-genes. Therefore, considering that *marT* is a functional gene in STM, but is a pseudogene in STY, that MarT positively regulates *misL* expression in STM, and that heterologous expression of *marT* negatively regulates *surV* expression in STY, the goals of this study are to search for and to characterize new genes regulated by MarT in *S. enterica* and to discover how pseudogenization of *marT* contributes to the evolution of *S. enterica* in becoming a pathogen restricted to human hosts.

## 2 Materials and methods

### 2.1 Cellular cultures and strains

All the strains used in this work were routinely cultured at 37°C with aeration in LB-medium (NaCl, 5 g/L; yeast extract, 5 g/L and peptone 10 g/L; prepared in phosphate buffer pH 7.0 all reagents obtained from Sigma-Aldrich). When needed, LB was solidified by adding agar 15 g/L (Sigma-Aldrich). For microaerophilic conditions, mineral oil was added on top of LB broth cultures and bacteria used to infect eukaryotic cells and mice.

### 2.2 *In silico* analyses

Using Basic Local Alignment Search Tool (BLAST) (Altschul et al., 1990), the consensus sequence TNAAANNNNNTNAAA was aligned against the *Salmonella Typhi* CT18 reference genome. The classical parameters were used, searching for a 100% match between the consensus sequence and the reference genome (considering the degenerated nucleotides N).

### 2.3 RNA isolation, reverse transcription, and real-time PCR

Total RNA from saturated bacterial cultures was extracted using phenol (Invitrogen) at 65°C. The nucleic acids were precipitated using absolute ethanol (Merck) at −80°C overnight and resuspended in nuclease-free water (Thermo Fisher Scientific). After the samples were treated with Turbo RNase Free DNase (Ambion) to remove the DNA, the RNA was quantified by spectrophotometry. Integrity of RNA was determined by electrophoresis using agarose gels (agarose 1% w/v; hypochlorite 1% v/v and ethidium bromide 0.5 μg/mL) with 500 ng per sample. Reverse transcription was performed using 1 μg of DNase-treated RNA with Superscript II RT (Invitrogen) at 50°C for 50 min. Relative quantification of each mRNA was performed using the Brilliant II SYBR Green QPCR Master Reagent Kit (Agilent). Real Time PCR was undertaken in a volume of 10 μL containing 1 μL of diluted cDNA (1:1000) and specific primers to detect each gene (Supplementary Table S1) in an Eco™ Real-Time PCR System (Illumina). Amplification efficiency was calculated from a standard curve constructed by amplifying serial dilutions of RT-PCR products. Normalization of expression was achieved against expression of 16S rRNA as described (Pfaffl, 2001; Hidalgo et al., 2016). Experiments were performed with at least three biological replicates with technical triplicates.

### 2.4 Construction of mutant strains

All mutants were derived from either *Salmonella enterica* serovar Typhi strain STH2370 or *Salmonella enterica* serovar Typhimurium strain 14028s. STY1408 mutant strains were obtained by facilitated allelic exchange using the Red-Swap method (Datsenko and Wanner, 2000). Briefly, 60 bp primers (Supplementary Table S1), whose 40 bp at the 5′ end exhibited 100% identity to target genes (STM14_2003 or STY1408), while the 20 bp at the 3′ end aligned to plasmids pKD3 or pKD4 were used to PCR amplify the cam or kan cassettes. Amplicons were electroporated into electrocompetent *S. typhimurium* ATCC14028s or *S. typhi* STH2370 harboring pKD46. Recombinant bacteria were selected on LB-agar + kan 50 μg/mL or LB-agar + cam 20 μg/mL. PCR amplification from colonies was carried out using primers RT-STY1408R + RT-STY1408F to verify mutations. Strains and their phenotypes are summarized in Table 1. In addition, a modification of the Red-Swap method (Datsenko and Wanner, 2000) was used to replace the STM14_2003 promoter with the ***tetRA*** cassette. Briefly, 60 bp primers (Supplementary Table S1), whose 40 bp at the 5′ end exhibited 100% identity to target gene STM14_2003, while the 20 bp at the 3′ end aligned to the ***terRA*** cassette present in the ***S**. typhimurium* 14028s ***yabB***:: ***tetRA*** mutant previously described (Hidalgo et al., 2004, 2016). Ones the STM14_2003 promoter was replaced with the ***tetRA*** cassette, ***tetA*** gene was replaced with the kan cassette as described above. Finally, the kan cassette was scised to produce a mutant containing the gene STM14_2003 under control of the tetracycline dependent promoter P^tetA^. We have successfully created this kind of genetic constructions previously (Millanao et al., 2020). Details of the production of this mutant and the promoter sequences interchanged can be found in Supplementary Figures S1, S2. kanamycin, chloramphenicol and tetracycline were obtained from Sigma.

**Table 1:** Strains used in this work.

Strain	Relevant genotype/characteristic	Source
*S. typhi* STH2370	Standard strain (WT).	Laboratory strain
*S. typhi* STH2370 ΔΨ*marT*::*marT*_STM_	Heterologously expressing *marT*_STM_	Laboratory strain
*S. typhi* STH2370 ΔSTY1408::*aph*	Deletional mutantion of gene STY1408, Kan^R^	This work
*S. typhimurium* 14028s	ATCC standard strain	ATCC
*S. typhimurium* 14028s Δ*marT*::*aph*	Deletional mutantion of gene *marT*, Kan^R^	Laboratory strain
*S. typhimurium* 14028sΔSTM14_2003::*cat*	Deletional mutantion of gene STM14_2003, Cam^R^	This work
*S. typhimurium* 14028s *mrmI*^TD^	STM 14028s ΔP*^mrmI^*::[*terR*-P*^tetA^-*FRT]-*mrmI,* expression of *mrmI* is tetracyclin dependent, Tet^S^	This work

Δ, gene frame deletion; Ψ, pseudogen; STM14_2003 (*S. typhimurium* 14028s) is homologous to STY1408 (*S. typhi* CT18), mrmI is the name suggested for STM14_2003 and STY1408. STY STH2370 is a standard strain extensively characterized (Santiviago et al., 2001; Hidalgo et al., 2004; Valenzuela et al., 2014).

### 2.5 Motility assay

Two microliter of bacteria grown to OD_600_ of 0.4, equivalent to 3×10^5^ CFU, were inoculated at the center of a Petri plates with 20 mL of semi-solid LB-agar (0.3% agar). The plates were dried for 30 min, incubated overnight at 37°C, and the diameter of bacterial growth was measured.

### 2.6 *In vitro* cell infection

The gentamicin protection protocol was carried out as previously described (Contreras et al., 1997). Briefly, bacteria were grown in microaerophilic conditions to OD_600_ of 0.2. Then, 5×10^4^ HEp2 cells (5×10^4^) were plated in each well of a 96-well plate using DMEM +10%FBS at 37°C and 5% CO_2_. Cells monolayers were infected next day with a bacteria-to-cell multiplicity of infection (MOI) of 100. One hour after infection, a group of cells monolayer were washed 3 times with 100 μL PBS and lysed in 100 μL of deoxycholate 0.5% p/v. Lysates were serially diluted and 5 μL plated on LB-agar. The plates were incubated for 16 h at 37°C before counting the CFUs. The same procedure was repeated for the other groups of cells infected, only that after the first hour of infection, the cells were maintained in medium with gentamicine at 50 μg/mL until lysis at 3 and 24 h postinfection. All culture medium, FBS, PBS and trypsin were acquired from Thermo Fisher Scientific. Plastic material was provided by Falcon.

### 2.7 *In vivo* infection

Microaerophilic cultures were grown as in the *in vitro* cell infection protocol, and 5×10^4^ bacteria were inoculated in each BALB/c mouse. In the case of orally infected mice, they were inoculated with a volume of 200 μL using a straight irrigation cannula (0.6 mm) with an oval tip on a tuberculin syringe. Orally infected mice were euthanized 5 days post-infection. In the case of Intraperitoneally infected mice, mice were inoculated with 100 μL using a tuberculin syringe (Cranberry). Intraperitoneally infected mice were euthanized 1 day post-infection. Weight of mice was measured each day during the assay. After sacrificed, mice were dissected to extract the liver and the spleen which were homogenized in 5 mL per every 1 g of tissue and 1 mL per every 0.1g of tissue, respectively. After serial dilution, 5 μL of each dilution was seeded on LB agar plates and incubated overnight at 37°C. Finally, CFU were counted to compare colonization of mutants versus wild-type (WT) strains. Experiments using the *S. typhimurium mrmI*^TD^ mutant were performed by oral infection as described above, with the addition that a group of mice started treatment with anhydrotetracycline (AHT, Thermo Fisher Scientific) at a concentration of 4 mg/mL in their drinking water. Experiments were conducted using protocols approved by the institutional bioethics committee at Universidad Andres Bello as described in the approval certificate 023/2015.

### 2.8 Statistical analyses

Two-tailed Student t-tests with α = 0.05 were employed, using GraphPad Prism 5.00.288.

## 3 Results

### 3.1 Identification of MarT target genes

The alignment of MarT binding sites (MTBS) in the STY CT18 reference genome identified 106 potential candidate target genes, all of which contain at least part of the canonical TNA_3_N_5_TNA_3_ sequence in their promoters. To filter and identify the best candidates, the promoters of confirmed MarT-target genes, such as *misL* and *surV*, were analyzed to define the most-characteristic parameters, such as position (distance from initial ATG), orientation, multiplicity, and whether the full or half of the consensus sequence is present (Supplementary Figure S3). In the *misL* promoter, two complete consensus sequences were found. The most-distal consensus sequence was located aproximately 360 nt upstream of the initial ATG of the *misL*-coding sequence (Supplementary Figure S3A). It is important to point out that although *in vitro* binding of MarT at the misL promoter is observed (along with biological effects after overexpressing misL) (Tükel et al., 2007), there is no significant expression under several conditions, as it can be found the the Salcom web site from Hinton’s Lab (Kröger et al., 2013).

For the *surV* promoter, only half MTBSs (TNAAA) were found 70, 110 and 140 nt upstream from initial GTG of the *surV*-coding sequence (Supplementary Figure S3B). Therefore, we searched for MTBS sequences in the forward orientation, while either the presence of a complete consensus sequence or just half of the consensus sequence were considered. After filtering the initial global search (Figures 1B,C), only 25 harbored MTBS located 350 upstream from the initial codon, yet only 4 presented two or more repeats (considering at least half the binding site). Thus, only 4 genes complied with all established MTBS parameters (Table 2 and Figure 1C). Out of the four genes identified, *mgtB*, *narQ*, and *acrR* have known functions (Snavely et al., 1991; Villagra et al., 2008), while the remaining gene (STY1408 and its STM homologous STM14_2003) have only been assigned putative functions based on sequence homology (Table 2).

**Table 2:** Putative MarT-target genes and their DNA-binding sequences found in the *S. typhi* CT18 genome.

Gene/sequence	Gene ID	Sequence^*^	Position	Function
Consensus MTBS	**–**	TNAAANNNNNTNAAA	**–**	Tükel et al. (2007)
*mgtB*	1,250,262	TaAAA	150	Transporter of magnesium
		TaAAAcccacTcAAA	145	
		TcAAA	72	
*acrR*	1,246,994	TcAAA	118	Repressor of *acrAB* genes
		TcAAAggtccTtAAA	103	
*narQ*	1,249,033	TAAAA	198	Putative sensor of nitrate
		TcAAAgcaaaTgAAA	47	
STY1408	1,247,802	TcAAA	81	Putative chemoreceptor
		TcAAAtgctaTaAAA	54	
		TaAAA	38	
		TcAAA	242	

*Lowercase within DNA-binding sequences indicates actual nucleotides corresponding to N in the consensus sequence. Position is relative to upstream distance from the first codon of the ORF.

**Figure 2:**
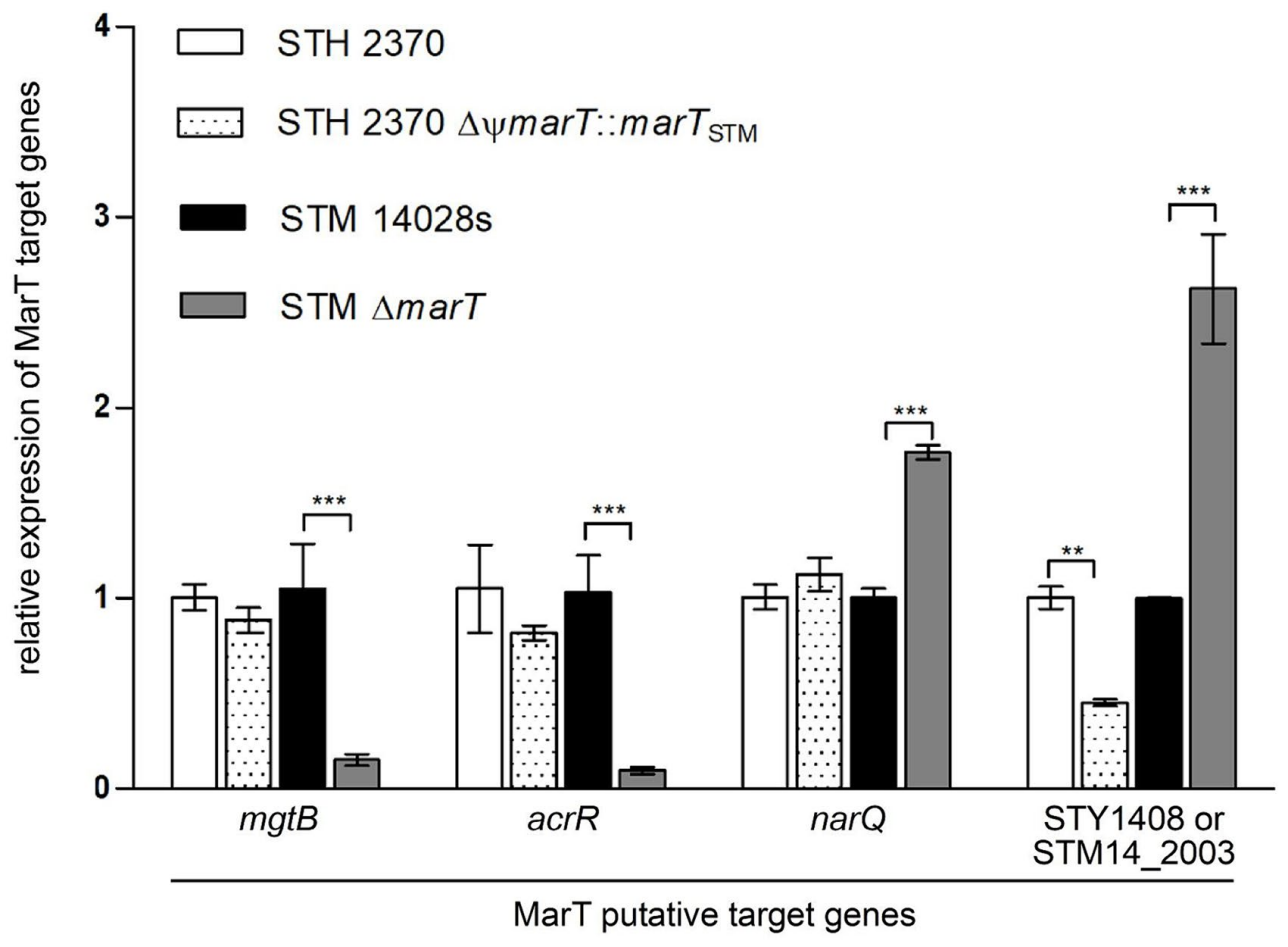
Quantitative PCR of putative MarT-target genes. Expression of the four genes selected was assessed in *S. typhi* after restoring *marT* with *marT*_STM_. Analogously, expression of the four genes was assessed in *S. typhimurium* after inactivating endogenous *marT*. A representative assay of 2 independent biological replicates is shown. ^**^p < 0.005, ^***^p < 0.0005.

To empirically confirm the regulation of the 4 final target candidates by MarT, their expression was studied in STM, STM Δ*marT*, STY STH2370 and STY STH2370 ΔΨ*marT*::*marT*_STM_. RNA isolated from stationary phase cultures was used to measure expression of genes *mgtB*, *acrR*, *narQ* and STY1408 (or its STM homologous gene STM14_2003). Expression of the four genes changed significantly in STM Δ*marT* compared with the STM WT strain (gray versus black bars in Figure 2). For genes *mgtB* and *acrR*, MarT upregulates their expression, as expression was significantly decreased in the MarT null mutant. For *narQ* and STM14_2003 (STM homologous of STY1408), downregulation by MarT was observed, as the *marT* deletion increased the expression of both genes. Analogously, expression of the four genes was examined in STY WT which does not express a full-length *marT* gene and was compared with expression in STY heterologously expressing *marT*_STM_. In STY, only STY1408 suffered changes in expression as a function of *marT* expression. In STY, the heterologous expression of *marT*_STM_ reduced expression of STY1408. This is consistent with the expression of STM14_2003 in STM which is diminished when *marT* is intact. In conclusion, a new gene downregulated by MarT in STM and STY was discovered, and the subsequent experiments were designed to understand the phenotypes associated with STY1408 and STM14_2003, in STY and STM, respectively.

### 3.2 Genetic and phenotypic characterization of STY1408 mutants

**Figure 3:**
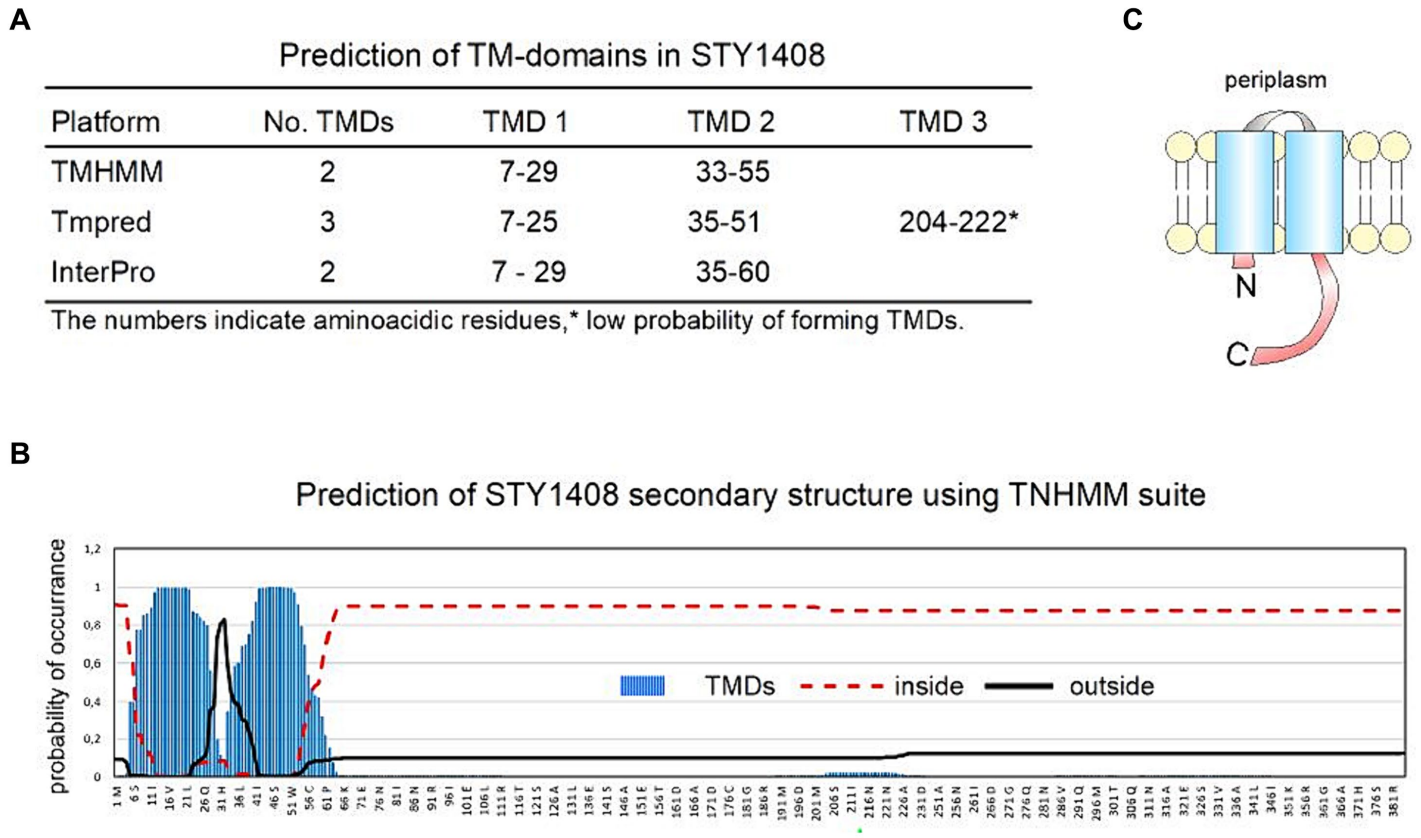
*In silico* sequence and structure analysis of the putative protein encoded by *mrmI* (STY1408). Sequence analysis predicts this gene encodes for a membrane protein, probably in the MCP family. **(A)** Three different programs (Sonnhammer et al., 1998; Ikeda et al., 2003; Blum et al., 2021) predict a membrane protein with 2 transmembrane domains (TMD). **(B)** Details of the probability of amino acid residues being located inside, outside or as part of a TMD according to TNHMM suite (Sonnhammer et al., 1998). **(C)** Topology of the predicted protein.

As mentioned above, the function of STY1408 is unknown, although the analyses of the nucleotide and predicted amino acid sequences indicate high identity and similarity with MCP-family chemosensors, such as Tsr, CheD and Aer. The identity, similarity and coverage of their sequences is summarized in Table 3. More details of aligned sequences can be found in Supplementary Figure S4, for both DNA-coding sequences and aminoacidic sequences. MCP stands for Methyl-accepting chemotaxis protein since proteins in this family participate in chemotaxis and have a methylated glutamate residue in the C-terminal domain. Biochemical and structural modeling studies suggest all proteins in the MCP-family form homodimers, although they might form superstructures by combining 2 or 3 dimeric structures (Derr et al., 2006; Alexander and Zhulin, 2007). This type of protein detects nutrients and other molecules to transduce signals that in turn modulate bacterial motility (Alexander and Zhulin, 2007). Prediction of transmembrane domains using three different on-line platforms indicate that STY1408 may span the inner membrane twice (Sonnhammer et al., 1998; Ikeda et al., 2003, p. 20; Blum et al., 2021) (Figure 3).

**Table 3:** Identity and similarity of STY1408 with sensor proteins.

Protein	Specie	Coverage	Identity	Similarity	Gaps
Tsr	*S. typhimurium* LT2	82	%38%	62	12^*^
CheD	*E. coli* K-12	78	40	64	0
Aer	*E. coli* K-12	80	36	54	6

^*^12 gaps are in the last 10 of aligned sequence at c-term. Coverage, identity, and similarity are in %. Clustal analysis of protein sequences and DNA-coding sequences are presented in Supplementary Figure S4.

**Figure 4:**
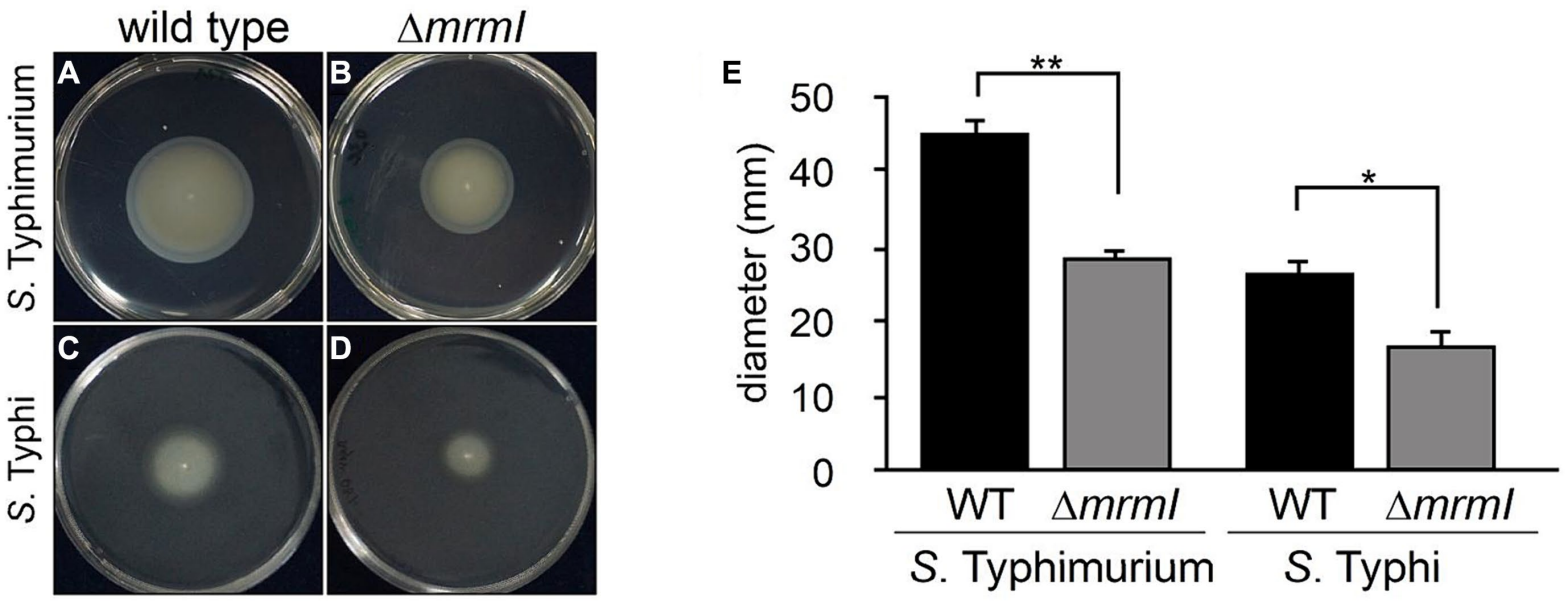
Soft-agar motility assay. The gene *mrmI* is involved in motility of *S. typhimurium*. Growth of STM and its derivative mutants on semi-solid agar plates. **(A–D)** show growth of STM WT, STM Δ*mrmI*, STY and STY Δ*mrmI*, respectively. **(E)** growth compared to WT strains measured as diameter in millimeters. *p < 0.05, **p ≤ 0.01. The figure shows a representative assay of 3 independent biological replicates, each with 3 technical replicates.

Therefore, we named STY1408 as “MarT-regulated motility gene I” or *mrmI*. Next, the motility of STM and STY mutants in the *mrmI* gene was evaluated. As shown in Figure 4, inactivation of *mrmI* reduced swimming in both serovars (by 40% for STY and by 20% for STM), when compared with their WT counterpart. The same assay was performed with strains STY Δ*marT* and STY STH2370 ΔΨ*marT*::*marT*_STM_; the results are consistent with *mrmI* regulation by MarT. Since MarT downregulates *mrmI* expression, inactivation of *marT* in STM resulted in increasing motility compared to STM WT, while heterologous expression of *mrmI* in STY produced a slight, yet not significant, reduction of motility (Supplementary Figure S5). With these observations, we conclude that *mrmI* is involved in motility, while *marT* is indirectly involved in this cellular function in STM.

### 3.3 The *mrmI* genes of *Salmonella* are required for infection of cells *in vitro*

**Figure 5:**
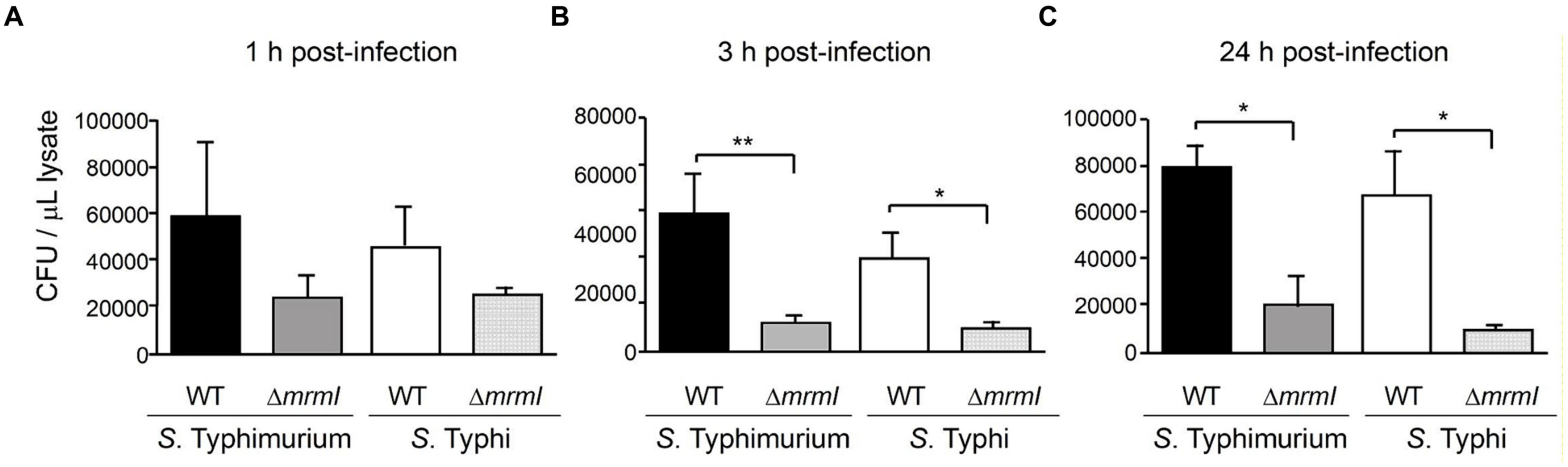
Gentamicin protection assay indicates *mrmI* mutants are defective in invasion and proliferation into cultured cells. Bacteria associated to cells 1- **(A)**, 3- **(B)** and 24-h **(C)** post-infection were assessed. In **(A)**, adherence is not significatively affected by deletion of *mrmI* in either STM or STY serovars. In **(B,C)**, defective invasion, and proliferation of *mrmI* mutants, inside cultured cells, is shown. Representative assays of 3 independent biological replicates are shown *p < 0.05, **p < 0.01.

As motility of *Salmonella* is directly involved in the process of infecting epithelial cells, the impact of inactivating *mrmI* on invasion was assessed. HEp-2 cells were infected with *mrmI* mutant strains of STM and STY and with their WT counterparts, to determinate whether *mrmI* is involved in the infective process (Olsen et al., 2013; Rivera-Chávez et al., 2013; Ryan et al., 2016). Interestingly, after 1 h of infecting the cells, STM and STY defective in *mrmI* presented lower (although statistically non-significant) association with HEp-2 epithelial cells, compared with their WT counterpart (Figure 5A). Impressively, after 3 h of infecting the cells, the association of STM and STY defective in *mrmI* was significantly impaired, compared with WT STM and WT STY, a feature that was also prevalent after 24 h (Figures 5B,C). Therefore, the results indicate that *mrmI* is involved in the process of infecting cells *in vitro*, in both STM and STY.

### 3.4 The *mrmI* gene of *Salmonella* is required to infect mice oraly

**Figure 6:**
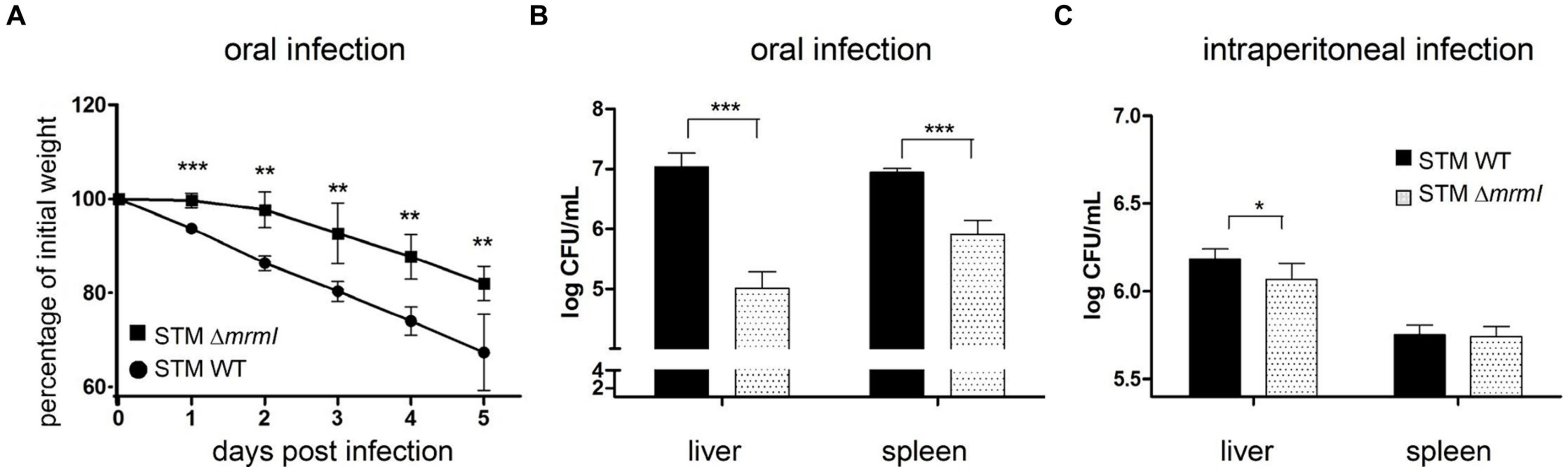
*In vivo* colonization of liver and spleen. Deletion of *mrmI* reduces virulence of *S. typhimurium* in orally infected mice. **(A)**, Weight evolution for 5 days after oral infection. **(B,C)**, CFU/mL of STM and STM Δ*mrmI* collected from oral and intraperitoneal infections, respectively, from liver and spleen. Note that the ordinate axes in panels **(B,C)** are in Log_10_ scale, in CFUx10,000. For each experiment, a representative assay of 3 biological independent replicates is shown. ^*^p < 0.05, ^***^p < 0.005.

**Figure 7:**
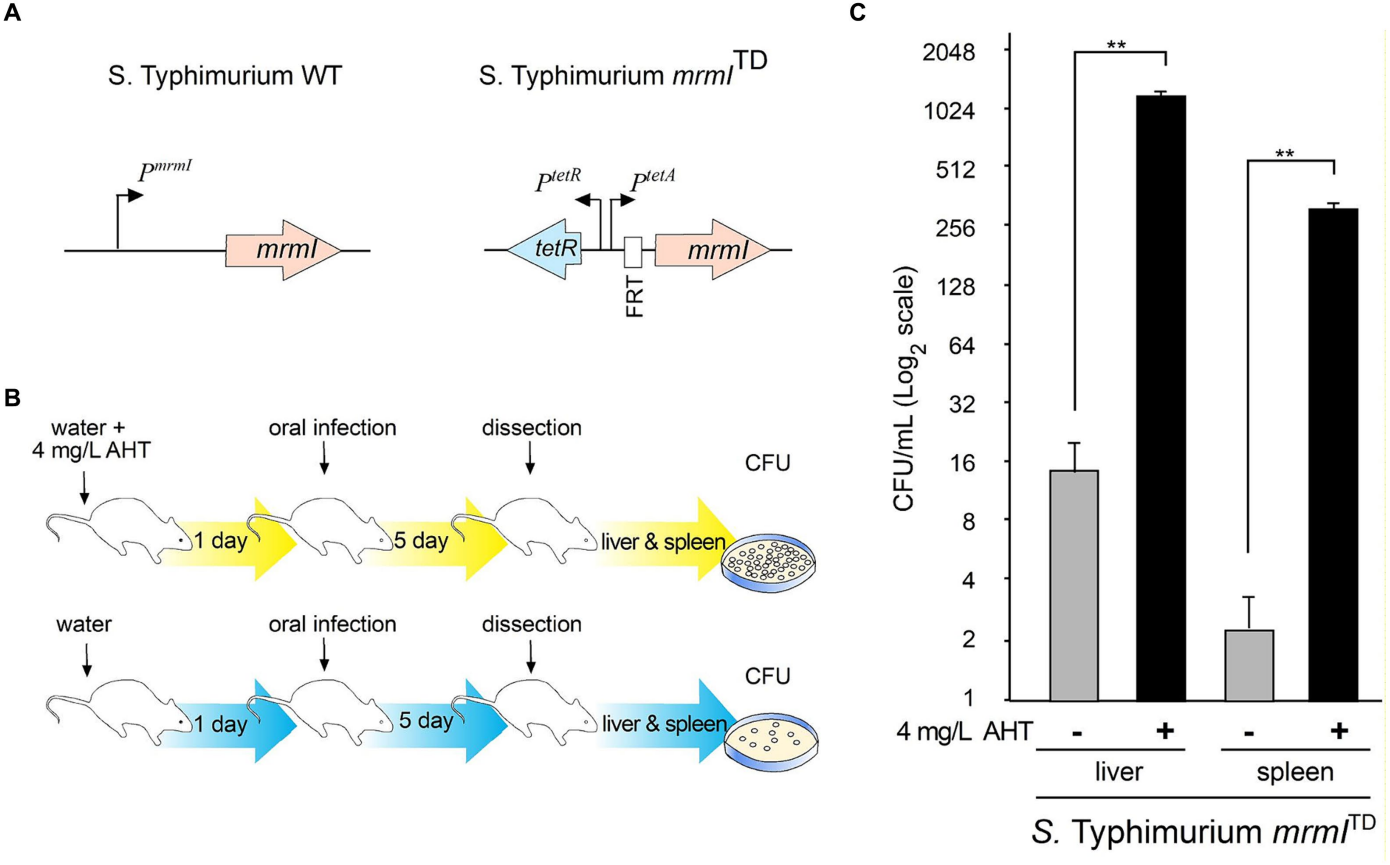
Complementation in cis of *mrmI* by using a tetracycline inducible system *in vivo*. The *S. typhimurium mrmI*^TD^ was used to orally infect mice maintained on a diet with AHT to assess the presence of bacteria in liver and mice. In **(A)**, details of the genetic construction that allow for expression of *mrmI* from the *tetA* promoter. In **(B)**, experimental setting to assess invasion of liver and spleen. In **(C)**, number of CFU in liver and spleen. The plot is in log_2_ scale to facilitate comparing induced and control conditions, in CFUx1,000. The experiment was performed twice with 3 mice each attempt. The results shown are representative from one experiment with technical triplicates. ^**^p < 0.01.

As shown above, *mrmI* is a new gene regulated by MarT that plays an important role in bacterial motility and *in vitro* infection of cells. For these reasons, we tested whether expression of *mrmI* is involved in the virulence of *Salmonella*, using the *in vivo* STM-mouse model of infection. Groups of mice were infected orally, and the weight of each animal was monitored daily for the next 5 days. Impaired virulence of STM Δ*mrmI* was observed, as less weight-loss was observed in mice infected with the mutant, compared with mice infected with STM WT; this trend was maintained throughout the 5 days of the experiment (Figure 6A). Day five post-infection, mice were euthanized, dissected, the liver and spleen macerated, and serially diluted suspensions plated on LB-agar to obtain CFU the next day. As shown in Figure 6B, STM Δ*mrmI* mutant was present in the liver at approximately 100 times lower levels compared to the WT strain. Similarly, STM Δ*mrmI* was present in the spleen at aproximately 10 times lower compared to the STM WT. However, when mice were infected intraperitoneally, deletion of *mrmI* only produced a marginal decrease in the infection of STM in liver and spleen (Figure 6C). To demonstrate the dependency of *mrmI* during the process of mice infection, oral infection was performed with the *S. typhimurium mrmI*^TD^ mutant (Figure 7A). In this mutant, transcription of *mrmI* depends on the presence of tetracycline. Therefore, a group of mice started treatment with anhydrotetracycline (AHT) at a concentration of 4 mg/mL in drinking water 24 h before oral infection. Mice were dissected 5 days after infection to study bacteria in the liver and spleen (Figure 7B). In the presence of AHT, bacterial counts in the liver and spleen were approximately 64 and 128 times higher, respectively, compared to control mice on a normal diet (Figure 7). This result indicates that the expression of *mrmI* increases deep organ invasion. Finally, mice were orally infected with STM Δ*marT*, to study the involvement of MarT in virulence of *Salmonella*. Day five post-infection, liver and spleen bacteria were collected and processed as before. Unexpectedly, STM Δ*marT* presented a less invasive phenotype (Supplementary Figure S6). Therefore, regulation of genes other than *mrmI*, might be responsible for the final phenotypes of *marT* null mutants. We conclude that *mrmI* plays a crucial role during invasion of STM via the mouse intestine.

## 4 Discussion

*In silico* analysis identified 4 candidate genes bearing at least one complete MarT-binding sequence in their promoter region. ‘The extension of the MarT-binding sequence (15 nt), its location, and its sometimes directly repeated nature suggest that MarT could act as a dimer to exert its function as a transcriptional regulator. In addition, the 10-nucleotide per turn DNA structure of Watson and Crick (Watson and Crick, 1953) is consistent with this idea. In fact, MarT belongs to the ToxR protein family, as it shares 32% amino acid similarity with ToxR (Blanc-Potard et al., 1999; Tükel et al., 2007). In *V. cholerae*, ToxR forms homodimers when overexpressed, while at normal expression levels, ToxR forms heterodimers with ToxS in the periplasm (Ottemann and Mekalanos, 1996). Furthermore, ToxR mutants with the inability to dimerize show defective DNA-binding capacity with downregulation of its regulon, in *V. cholerae* (DiRita and Mekalanos, 1991) reinforcing the idea that MarT could exert its function as a dimer.

MarT possesses a DNA binding domain with a winged helix-turn-helix. MarT regulates expression of *surV* and might bind to regulatory sequences by recognizing heptameric direct repeats present in the *surV* promoter, as is the case of ToxR regulation of the cholera toxin, which recognizes the heptameric sequence TTTTGAT (Miller et al., 1987). Other proteins in the ToxR family recognize sequences different to MarT-binding sequences (Tükel et al., 2007). Such is the case of TcpP, which recognizes the sequence TGTAANNNNNNTGTAA (Goss et al., 2010) and PhoB which targets the TGTCANNNNNNTGTCA (Diniz et al., 2011). Thus, we speculate that the consensus MarT-binding sequence might differ from the consensus for ToxR-binding sequences. MarT-binding sequences, full direct repeats, and half direct repeats are located upstream from *mgtB*, *acrR*, *narQ* and *mrmI*. However, MarT is a positive regulator of *mgtB* and *acrR*, but a negative regulator of *narQ* and *mrmI* (Figure 2). How binding site structure, interactions and topology affect MarT as a negative or positive regulator, is unknown. The heterologous expression of *marT*_STM_ in STY adds another level of complexity to understanding the effects of MarT, as it acts as a positive regulator only of *mrmI*, with no significant effect on transcription of *mgtB*, *acrR* or *narQ*.

The *mrmI* gene has 98% identity with members of the MCP-protein family, which are implicated in the sensing of sugars and motility. Deficient motility was observed in *mrmI* mutants of both STM and STY, possibly determined by a defective detection of sugars, which is characteristic of the chemotaxis function of MCPs (Lux et al., 1995). MCPs sense sugars including maltose, ribose and galactose (Parkinson, 1976).

Reports show motility or chemotaxis related genes are essential for the STM infective cycle, since their participation in the colonization process is crucial, both *in vitro* and *in vivo.* This was shown as mutants in motility and chemotaxis related genes are less invasive (Olsen et al., 2013). In fact, a study in *S. enterica* serovar Enteritidis shows that pathogenic strains express higher levels of virulence transcriptional regulators and virulence-involved genes, such as motility and fimbriae genes, compared to Low-Pathogenic strains (Barbosa et al., 2017).

The result of *in vitro* infection indicates that both serovars Typhi and Typhimurium defective in *mrmI* gene presented a poor association with cultured cells 1, 3 and 24 h post infection. Further characterization of *S. typhimurium mrmI* in the murine infection model revealed that *mrmI* is important in the invasion via the intestinal barrier, as only orally infected mice showed important defects in liver and spleen invasion. On the other hand, colonization of liver and spleen changed only slightly after intraperitoneal infection with *S. typhimurium mrmI*. In addition, the preliminary results of intraperitoneally infected mice with 1,000 CFU of *Salmonella Typhi*murium *mrmI* show no significant differences in liver and spleen invasion compared to the wild-type counterpart 5 days post-infection. These results highlight the importance of *mrmI* and chemotaxis for invading and crossing the intestinal barrier. In support of these findings, it has been reported that motility genes such as flagellar structural components, flagellar synthesis machinery and flagellar export components, play no part in systemic infections of STM in mice macrophages (Schmitt et al., 2001) Nevertheless, colonization of organs other than liver and spleen via different administration routes requires further analysis. Therefore, we propose that *mrmI* participates in intestinal phase of infection, but it loses importance during the systemic cycle, where the bacteria are phagocytized and disseminated by phagocytic cells (Hurley et al., 2014).

Given that *mrmI* is involved in motility of STM and STY and that *mrmI* expression is regulated (directly or indirectly) by MarT, it was found that MarT is involved in motility, as the *S. typhimurium* Δ*marT* mutant displayed increased motility (Supplementary Figure S5). Therefore, MarT might regulate motility at least in part by negatively regulating *mrmI*. Our results indicate that MarT is a negative regulator of *mrmI*, and for this reason, we predicted that deletion of *marT* would increase expression of *mrmI* and increase invasion, to liver and spleen, as a consequence. However, STM Δ*marT* presented a less invasive phenotype, compared to STM WT in mice (Supplementary Figure S6). Therefore, regulation of genes other than *mrmI*, might be responsible for the final phenotypes of *marT* mutants. Studying the regulon of *marT* by using global search approaches will elucidate the contribution of other genes to final phenotypes of *marT* mutants.

As mentioned above, MarT belongs to the ToxR family protein (Blanc-Potard et al., 1999; Tükel et al., 2007). These proteins are bound to the inner membrane of Gram-negative bacteria, where they sense particular compounds and conditions to produce a genomic response (Auger et al., 1989; DiRita and Mekalanos, 1991). One example is CadC, which harbors 41% identity with MarT (Blanc-Potard et al., 1999). At pH 5.8 and a rich lysine environment, CadC positively regulates the operon *cadBA* in *E. coli* (Auger et al., 1989). *cadBA* encodes proteins that responsible for lysine catabolization and the expulsion of cadaverine resulting from lysine decarboxylation (Supplementary Figure S7) (Dell et al., 1994; Cserzö et al., 1997; Tetsch et al., 2008). *In silico* analyses of MarT reveal a transmembrane domain (Supplementary Figure S7) suggesting that MarT might act through mechanisms similar to those used by CadC.

## 5 Conclusion

According to our results, we conclude that MarT is involved in downregulating expression of *mrmI* in STM, while *marT* is pseudogenized in STY. In addition, decreased expression of *mrmI* when *marT*_STM_ is restored in STY further supports the evidence that MarT downregulates *mrmI*. Furthermore, it was found that *mrmI* possesses homology and high levels of similarity with methyl accepting chemotaxis proteins such as Tsr, CheD, and Aer. the *mrmI* gene is involved in the motility of both STM and STY and might play a crucial role in host invasion through the intestinal barrier. We speculate that STY evolved in such a way to silently cross the intestinal barrier to avoid alerting the immune system, thus favoring systemic invasion.

## Data availability statement

The datasets generated for this study are available on request from the corresponding author, AH.

## Ethics statement

The animal study was approved by institutional bioethics committee at Universidad Andres Bello. The study was conducted in accordance with the local legislation and institutional requirements.

## Author contributions

SJ: Conceptualization, Data curation, Formal analysis, Investigation, Validation, Writing – original draft, Methodology, Software. ArM: Data curation, Formal analysis, Investigation, Methodology, Resources, Validation, Writing – review & editing. AnM: Methodology, Writing – review & editing, Formal analysis, Investigation, Resources. CS: Data curation, Methodology, Writing – review & editing, Validation, Visualization. SB: Formal analysis, Investigation, Resources, Writing – review & editing, Data curation, Methodology. GM: Resources, Supervision, Writing – review & editing, Funding acquisition, Methodology, Project administration. NV: Conceptualization, Data curation, Formal analysis, Project administration, Resources, Supervision, Writing – review & editing. AH: Conceptualization, Data curation, Formal analysis, Investigation, Validation, Writing – review & editing, Funding acquisition, Project administration, Resources, Supervision, Visualization, Writing – original draft.
