# Supplementary material for: Transcriptional regulator MarT negatively regulates MarT-regulated motility gene I, a new gene involved in invasion and virulence of Salmonella enterica
Source: Front Microbiol. 2024 Aug 15;15:1430982. doi: 10.3389/fmicb.2024.1430982 (PMC11358694; doi:10.3389/fmicb.2024.1430982)
Supplement: Supplementary file 1 [file Data_Sheet_1.docx]

Supplementary Material

# Supplementary Figures and Tables

## Supplementary Figures


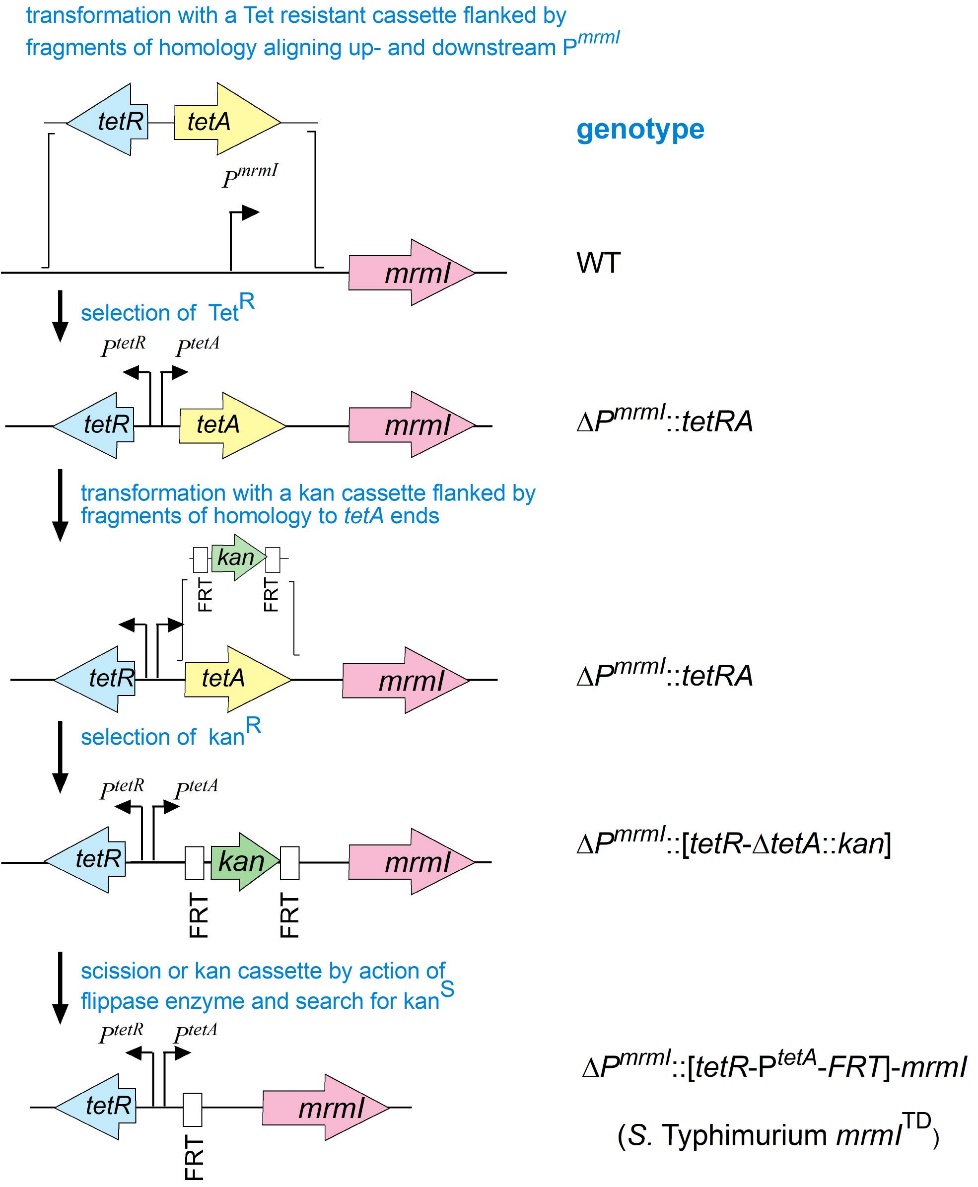
Supplementary Figure 1

**Supplementary Figure 1**. Schematic representation of the steps to obtain *S.* Typhimurium *mrmI* ^TD^. The mutant was obtained by performing facilitated allelic interchange. The first step involves a modification of the protocol originally reported by Datsenko and Wanner (2000). In this case, the *tetRA* cassette was amplified from a chromosomal insertion of the *tetRA* cassette in the *yabB* gene. The *yabB* insertion produces tetracycline-dependent growth and allows to avoid unwanted selection of the insertion of the template DNA by simple streaking of mutants with and without tetracycline. After replacing the *tetA* gene with the kan cassette using the traditional red-swap technique, the kan cassette is excised by the action of the Flippase enzyme.

Supplementary Figure 2

A) Promoter region of *mrmI* of STM 14028s

TATGCCTGATATAACGCCTCATCCAAACGCTCAGGATTCTGCAAGAGAACGGGCGATAACGCTGTCCGCGGTAATAACACGGAGAAACAGTCATTCGCTTATTTAAGCGTCTAT**TAAAA**TATACTGGCTTTCGACTTAACTATTAAGCAGAAAATGGTGATAAGTACATCACTTGTCAATATGGATGGATGAATATCCTGGCATAGCGAATATAGATGATTTTATCTGTTGCACTAAGGGAAATGGCGATGGTCTGGTATTGAATATGGAGAATGTCACAGAAACGAATGGCAATAGGTATC**TGAAAtgctaTAAAA**A**TAAAA**CCTGTCTCCTATCCTGGTTGT**AGGCCGT**CAAAA**ATG**TTGAGAAATATCAGCGTCAGGACATGCATTATTCTATTCATGGTA…

B) Promoter region of *mrmI*(STY1408) STY CT18

GAGAAACAGTCATTCACTTATTTAAGCGTCTAT**TAAAA**TATACTGGCTTTCGACTTAACTATTAAGCAGAAAATGGTGATAAGTACATCACTTGTCAATATGGATGGATGAATATCCTGGCATAGCGAATATAGATGATTTTATCTATTGCACTAAGGGAAATGGCGATGGTCTGGTATTGAATATGGAGAATG**TCAAA**GAAACGAATGGCAATAGGTATC**TGAAAtgctaTAAAA**A**TAAAA**CCTGTCTCCTATCCTGGTTGT**AGGCCGT**CAAAA**ATG**TTGAGAAATATCAGCGTCAGGACATGCATTATTCTATTCATGGTA…

C) sequence of T-POP transposon, containing the *tetRA* cassette

CTGATGAATCCCCTAATGATTTTATCAAAATCATTAAGGTTACCATCACGGAAAAAGGTTATGCTGCTTTTAAGACCCACTTTCACATTTAAGTTGTTTTTCTAATCCGCATATGATCAATTCAAGGCCGAATAAGAAGGCTGGCTCTGCACCTTGGTGATCAAATAATTCGATAGCTTGTCGTAATAATGGCGGCATACTATCAGTAGTAGGTGTTTCCCTTTCTTCTTTAGCGACTTGATGCTCTTGATCTTCCAATACGCAACCTAAAGTAAAATGCCCCACAGCGCTGAGTGCATATAATGCATTCTCTAGTGAAAAACCTTGTTGGCATAAAAAGGCTAATTGATTTTCGAGAGTTTCATACTGTTTTTCTGTAGGCCGTGTACCTAAATGTACTTTTGCTCCATCGCGATGACTTAGTAAAGCACATCTAAAACTTTTAGCGTTATTACGTAAAAAATCTTGCCAGCTTTCCCCTTCTAAAGGGCAAAAGTGAGTATGGTGCCTATCTAACATCTCAATGGCTAAGGCGTCGAGCAAAGCCCGCTTATTTTTTACATGCCAATACAATGTAGGCTGCTCTACACCTAGCTTCTGGGCGAGTTTACGGGTTGTTAAACCTTCGATTCCGACCTCATTAAGCAGCTCTAATGCGCTGTTAATCACTTTACTTTTATCTAATCTAGACAT**CAT**TAATTCCTAATTTTTGTTGACACTCTATCATTGATAGAGTTATTTTACCACTCCCTATCAGTGATAGAGAAAAGTGAA**ATG**AATAGTTCGACAAAGATCGCATTGGTAATTACGTTACTCGATGCCATGGGGATTGGCCTTATCATGCCAGTCTTGCCAACGTTATTACGTGAATTTATTGCTTCGGAAGATATCGCTAACCACTTTGGCGTATTGCTTGCACTTTATGCGTTAATGCAGGTTATCTTTGCTCCTTGGCTTGGAAAAATGTCTGACCGATTTGGTCGGCGCCCAGTGCTGTTGTTGTCATTAATAGGCGCATCGCTGGATTACTTATTGCTGGCTTTTTCAAGTGCGCTTTGGATGCTGTATTTAGGCCGTTTGCTTTCAGGGATCACAGGAGCTACTGGGGCTGTCGCGGCATCGGTCATTGCCGATACCACCTCAGCTTCTCAACGCGTGAAGTGGTTCGGTTGGTTAGGGGCAAGTTTTGGGCTTGGTTTAATAGCGGGGCCTATTATTGGTGGTTTTGCAGGAGAGATTTCACCGCATAGTCCCTTTTTTATCGCTGCGTTGCTAAATATTGTCACTTTCCTTGTGGTTATGTTTTGGTTCCGTGAAACCAAAAATACACGTGATAATACAGATACCGAAGTAGGGGTTGAGACGCAATCGAATTCGGTATACATCACTTTATTTAAAACGATGCCCATTTTGTTGATTATTTATTTTTCAGCGCAATTGATAGGCCAAATTCCCGCAACGGTGTGGGTGCTATTTACCGAAAATCGTTTTGGATGGAATAGCATGATGGTTGGCTTTTCATTAGCGGGTCTTGGTCTTTTACACTCAGTATTCCAAGCCTTTGTGGCAGGAAGAATAGCCACTAAATGGGGCGAAAAAACGGCAGTACTGCTCGGATTTATTGCAGATAGTAGTGCATTTGCCTTTTTAGCGTTTATATCTGAAGGTTGGTTAGTTTTCCCTGTTTTAATTTTATTGGCTGGTGGTGGGATCGCTTTACCTGCATTACAGGGAGTGATGTCTATCCAAACAAAGAGTCATCAGCAAGGTGCTTTACAGGGATTATTGGTGAGCCTTACCAATGCAACCGGTGTTATTGGCCCATTACTGTTTGCTGTTATTTATAATCATTCACTACCAATTTGGGATGGCTGGATTTGGATTATTGGTTTAGCGTTTTACTGTATTATTATCCTGCTATCGATGACCTTCATGTTAACCCCTCAAGCTCAGGGGAGTAAACAGGAGACAAGTGCTTAGTTATTTCGTCACCAAATGATGTTATTCCGCGAAATATAATGACCCTCTTGATAACCCAAGAGGGCATTTTTTACGATAAAGAAGATTTATGTAGGCATCAATCATAATTAATTGCTGCTTATAACAGGCACTGAGTAATTGTTTTTTATTTTTAAAGTGATGATAAAAGGCACCTTTGGTCACCAACGCTTTTCCCGAGATCATATGACAAGATGTGTATCCACCTTAACTTAATGATTTTTACCAAAATCATTAGGGGATTCATCAG

**Supplementary Figure 2.** Promoter region of *mrmI* from *S.* Typhimurium LT2 (A), from *S.* Typhi (B), and the *tetRA* cassette from the **miniTn*10*dtet** transposon, T-POP (C). For (A) and (B), the coding sequences are shown in green, with their initial ATG in bold. The Shine-Dalgarno sequence, AGGCCGT, is in bold black and located 5 nt upstream from the initial ATG. The sequences included in the primers H1 mrmI-tetR and H2 mrmI-tetA, used to amplify the *tetRA* cassette to replace P*^mrmI^* with the *tetRA* cassette, are underlined. Putative MTBS are highlighted in blue. For (C), the *tetR* and *tetA* coding sequences are shown in light and dark green, respectively. The sequences included in the primers H1 mrmI-tetR and H2 mrmI-tetA are underlined. The inverted-repeat (IR) sequences of Tn*10* are highlighted in purple. Complete sequences of primers H1 mrmI-tetR and H2 mrmI-tetA are shown in **Supplementary Table 1**.

Supplementary Figure 3

A) *misL* *S*. Typhimurium LT2

TATCTTTGTTGATTTTTCTTTCCCGAATCTTCGACGATTTACCGATTTGCAATGGGCTGACAGTCTGGCAGACAGTGGCATGCATATTGTCCTGATTTCTGACAGAAGTTTAACTCCGCTGGCCAATTACTGGATTT**TGAAAagcaaTAAAA**TACAAGGGATTATTTATTCCGATGACGATGATATTGTGCAACAGCAAAAGATGCACCGCTTGTTTACCGGTCGGCTCGCCAACAGTAAACGTGGACGAACGCTTAACTATACGGAATTTATATTACTGAAGCGCTTTGTATCAGGAATAAGTATTCAGCAGATTGTAAATATTGACAATATTGATATTAAGAAACTTTATGTGCATAAGCTGCGGCTTGAAAATAAGCTAGGGCATAGCATTCA**TAAAAtaataTCAAA**TATTTTATAGATCCGTTTCCATTTTTATTATTTCCATATTATTGCGATGTGGAAGACGCTTTACGCCATAATGC**AGGAGG**CAGA**ATG**CCAACTCCCCAAAATTACTCATTCATCGCCATAGCGGTATCAGCAGCATTAGCCTCTATGGTATTTC…

B) *surV*(t3766) *S.* Typhi Ty2

GGTACGGGCTCACATCATAAGGGTGGATGGTGTTCATACGAGCAGTAACGTCGCAGCTTACTCATCTACTTGAAGGCACTATAGCCTTACGTTTATTAACGCAGGCGCTACGTTTGTCGATGCCGTAACTTTC**TGAAA**GGGAAGGCACAATGAGGCATGTGA**TCAAAA**CTCGCCGGGGACAGATGCCTTACTCACTGCAAA**TGAAAA**TCCACCACAAGATTCGGATCAATCGACCCGTCGCTGGCGAAACTTTAGACGAGACAA**AGCAGA**TTTA**GTG**ACGCTTCTGCTCAATGAGCAATATCATCTTTGCTGTTACAGCGAA…

**Supplementary Figure 3**. MTBS analysis in promoters of known MarT-target genes. (A) *misL* and (B) *surV*. The coding sequences are shown in green, with their initial ATG or GTC in bold. The Shine-Dalgarno sequence are in bold black and located 4 nt upstream from the fist codon. The MTBS sequences are highlighted in blue, within the promoter region. The sequences of *misL* and *surV* (t3766) were analyzed using Vector NTI 10.3.2 software and presented in word format.

Supplementary Figure 4

A) CLUSTAL Omega, multiple sequence alignment: Protein

Aer-Ecoli -MSSHPYVTQQNTPLADDTTLMSTTDLQSYITHANDTFVQVSGYTLQELQGQPHNMV--R 57

mrmI-STy ------------------------------------------------------------ 0

mrmI-STm ------------------------------------------------------------ 0

Tsr-STm MLKRIKIV----------TSLLLVLALFGLL------QLTSGGLFFNSLKNDKENFTVLQ 44

Tsr-Ecoli MLKRIKIV----------TSLLLVLAVFGLL------QLTSGGLFFNALKNDKENFTVLQ 44

Aer-Ecoli HPDMPKAAFADMWFTLKKG--------EPWS--------G-----I---VKNRRKNGDHY 93

mrmI-STy ------------------------------------------------------------ 0

mrmI-STm ------------------------------------------------------------ 0

Tsr-STm TIRQQQSALNATWVELLQTRNTLNRAGIRWMMDQSNIGSGATVAELMQGATNTLKLTEKN 104

Tsr-Ecoli TIRQQQSTLNGSWVALLQTRNTLNRAGIRYMMDQNNIGSGSTVAELMESASISLKQAEKN 104

Aer-Ecoli WVRANAVPMVREGKISGYMSIRTRATDEEIAAVEPLYKALNAGRTSKRIHKGLV-----V 148

mrmI-STy -----------------------------------MLRNIS------------------V 7

mrmI-STm -----------------------------------MLINIS------------------V 7

Tsr-STm WEQYEALPRDPRQSEAAFLEIKR-TYDIYHGALAELIQLLGAGKINEFFDQPTQSYQDAF 163

Tsr-Ecoli WADYEALPRDPRQSTAAAAEIKR-NYDIYHNALAELIQLLGAGKINEFFDQPTQGYQDGF 163

: :. .

Aer-Ecoli RKGWLGKLPSLPLRWRARGVMTLMFILLAAMLWFVAAPVVTYILCALVVLLASACFEWQI 208

mrmI-STy RTCIILFMVC-TF----LLVDT-LQITFLHDLPILITCNIIYLISALLLWWYMTC---YL 58

mrmI-STm RTCIILFMVC-AF----LLVDT-LQIAFLHDLPILITCNIIYLISALLLWWYMTC---YL 58

Tsr-STm EKQYMAYMQQ-NDRLYDIAVED-NNSSYNQAMWVLVSVLIAVLVVIIAVWFGIKL---SL 218

Tsr-Ecoli EKQYVAYMEQ-NDRLHDIAVSD-NNASYSQAMWILVGVMIVVLAVIFAVWFGIKA---SL 218

.. : : * : .: : : : : :

Aer-Ecoli VRPIENVAHQALKVATGERNSVEHLNRSDELGLTLRAVGQLGLMCRWLINDVSSQVSSVR 268

mrmI-STy VVPINTAKKSIEEVAAGNLSIHISEFGNNCAGRLIPGINSLSENISALVREIRSSSQTAM 118

mrmI-STm VVPINTVKKSIEEVAAGNLSIHISEFGNNCAGRLIPGINSLSENISALVREIRSSSQTAM 118

Tsr-STm IAPMNRLIESIRHIASGDLVKRIDVEGSNEMGQLAENLRHMQSELMRTVGDVRNGANAIY 278

Tsr-Ecoli VAPMNRLIDSIRHIAGGDLVKPIEVDGSNEMGQLAESLRHMQGELMRTVGDVRNGANAIY 278

: *:: .. .:* *: .: * : : : :: . .:

Aer-Ecoli NGSETLAKGTDELNEHTQQTVDNVQQTVATMNQMAASVKQNSATASAADKLSITASNAAV 328

mrmI-STy TLSEQLAARSLSLSVKTEQQSASLIQTAASMDEMAASTKNNADNTRMASIQADCATQCAR 178

mrmI-STm TLSEQLAARSLSLSVKTEQQSASLIQTAASIDEMAASTKNNADNTRMASIQADCATQCAR 178

Tsr-STm SGASEIAMGNNDLSSRTEQQAASLEETAASMEQLTATVKQNAENARQASHLALSASETAQ 338

Tsr-Ecoli SGASEIATGNNDLSSRTEQQAASLEETAASMEQLTATVKQNAENARQASHLALSASETAQ 338

. :. :* . .*. :*:* .: :*.*::::::*:.*:*: .: *. : *:: *

Aer-Ecoli QGGEAMTTVIKTMDDIADSTQRIGTITSLINDIAFQTNILALNAAVEAARAGEQGKGFAV 388

mrmI-STy KGGELMVRVTENMRSITDCASQMTEIISLIDGIAFQTNILALNAAVEAARAGDHGKGFSV 238

mrmI-STm KGGELMVRVTENMRSITDCASQMTEIISLIDGIAFQTNILALNAAVEAARAGDHGKGFSV 238

Tsr-STm KGGKVVDNVVQTMRDIASSSQKIADIISVIDGIAFQTNILALNAAVEAARAGEQGRGFAV 398

Tsr-Ecoli RGGKVVDNVVQTMRDISTSSQKIADIISVIDGIAFQTNILALNAAVEAARAGEQGRGFAV 398

:**: : * :.* .*: .:.:: * *:*:.********************::*:**:*

Aer-Ecoli VAGEVRHLASRSANAANDIRKLIDASADKVQSGSQQVHAAGRTMEDIVAQVKNVTQLIAQ 448

mrmI-STy VAREVRNLAHRSAEAAKNIKALIDVTHDNVRQGAAIVQEAEKNMQEIVGGSGQLNVLMSE 298

mrmI-STm VAGEVRNLAHRSAEAAKSIKALIDVTHDNVRQGAAIVQEAEKNMQEIVGGSGQLNVLMSE 298

Tsr-STm VAGEVRNLAQRSAQAAREIKSLIEDSVSRVDVGSTLVESAGETMDEIVNAVTRVTDIMGE 458

Tsr-Ecoli VAGEVRNLAQRSAQAAREIKSLIEDSVGKVDVGSTLVESAGETMAEIVSAVTRVTDIMGE 458

** ***:** ***:**..*: **: : ..* *: *. * ..* :** .:. ::.:

Aer-Ecoli ISHSTLEQADGLSSLTRAVDELNLITQKNAELVEESAQVSAMVKHRASRLEDAVTVLH-- 506

mrmI-STy ISTTTREQEKGINQITLALSDLESATHSNVLMVEALSASSDVLKAQVIELQTKTDKFRLS 358

mrmI-STm ISTTTREQEKGINQITLALSDLESATHSNVLMVEALSASSDVLKAQVIELQTKTDKFRLS 358

Tsr-STm IASASDEQSRGIDQVGLAVAEMDRVTQQNASLVEESAAAAAALEEQASRLTQAVAVFRIH 518

Tsr-Ecoli IASASDEQSRGIDQVGLAVAEMDRVTQQNAALVEESAAAAAALEEQASRLTEAVAVFRIQ 518

*: :: ** *:..: *: ::: *:.*. :** : : :: :. .* . ::

Aer-Ecoli ------------------------------------ 506

mrmI-STy QPGYSEHALSRSHVSPLSTITRR-----GQA----- 384

mrmI-STm QPGYSEHALSHSHVSSLSTITRR-----GQA----- 384

Tsr-STm QQQQRAREVAAV-KTPAAVSSPKAAVADGSDNWETF 553

Tsr-Ecoli QQQRETSAVVKT-VTP--AAPRKMAVADSEENWETF 551

B) CLUSTAL Omega multiple sequence alignment: DNA

Aer-E.coli -------------------------------------------------ATGTCTTCTCA 11

mrmI-STy ------------------------------------------------------------ 0

mrmI-STm ------------------------------------------------------------ 0

Tsr-Stm GCGGAAAGAGAAAACATGTTAAAGCGAATTAAAATTGTTACCAGCTTACTGCTGGTATTG 60

Tsr-E.coli ---------------ATGTTAAAACGTATCAAAATTGTGACCAGCTTACTGCTGGTTTTG 45

Aer-E.coli TCCGTATGTCACCCAGCAAAATACCCCGCTGGCGGACGATACCACTCTGATGTCCACTAC 71

mrmI-STy ------------------------------------------------------------ 0

mrmI-STm ------------------------------------------------------------ 0

Tsr-Stm GCGCTATTTGGCCTTTTACAACTGACATCCGGCGGGCTGTTCTTCA-----ACTCGCTGA 115

Tsr-E.coli GCCGTTTTTGGCCTTTTACAACTGACATCAGGCGGTCTGTTCTTTA-----ATGCCTTAA 100

Aer-E.coli CGATCTGCAAAGCTATATCACTCATGCTAATGACACTTTTGTGCAGGTGAGCGGCTATAC 131

mrmI-STy ------------------------------------------------------------ 0

mrmI-STm ------------------------------------------------------------ 0

Tsr-Stm AGAATGACAAAGAAAACTTCACCGTATTGCAAACTATTCGTCAGCAGCAGTCTGCCCTGA 175

Tsr-E.coli AGAATGACAAAGAAAATTTCACTGTTTTACAAACCATTCGCCAGCAGCAATCCACGCTGA 160

Aer-E.coli CTTGCA----AGAGTTACAAGGGCAGCCGCACAACATGGTGCGTCACCCGGATATGCCAA 187

mrmI-STy ------------------------------------------------------------ 0

mrmI-STm ------------------------------------------------------------ 0

Tsr-Stm ATGCAACCTGGGTGGAGCTGTTGCAAACGCGTAATACCCTGAATCGCGCGGGTATCCGCT 235

Tsr-E.coli ATGGCAGCTGGGTCGCGTTGTTGCAGACGCGTAACACCCTCAACCGCGCGGGTATCCGCT 220

Aer-E.coli AAGCGGCGTTTGCGGAT-ATGTGGTTCACCCTGAAAAAAGGGGAGCCCTGGAGCGGCATC 246

mrmI-STy ------------------------------------------------------------ 0

mrmI-STm ------------------------------------------------------------ 0

Tsr-Stm GGATGATGGACCAGAGCAATATTGGCAGCGGCGCAACTGTCGCTGAACTGATGCAGGGGG 295

Tsr-E.coli ACATGATGGATCAGAATAATATTGGTAGCGGTTCAACCGTTGCTGAGCTGATGGAGAGTG 280

Aer-E.coli GTGAAAAATCGCCGCAAAAATGGTGACCATTATTGGGTGCGGGCCAATGCGGTACCGATG 306

mrmI-STy ------------------------------------------------------------ 0

mrmI-STm ------------------------------------------------------------ 0

Tsr-Stm C-GACCAATACGCTGAAGCTGACCGAAAAAAACTGGGAGCAGTATGAGGCGTTACCGCGC 354

Tsr-E.coli C-CAGTATTTCGCTGAAACAGGCGGAAAAAAACTGGGCGGATTACGAAGCGTTGCCGCGT 339

Aer-E.coli GTGCGCGAGGGAAAAATCAGTGGCTATATGTCGATTCGTACCCGGGCGACGGATGAAGAG 366

mrmI-STy ------------------------------------------------------------ 0

mrmI-STm ------------------------------------------------------------ 0

Tsr-Stm GATCCA-CGTCAGAGTGAAGCGGCTTTCCTTGAGATC--AAACGAACCTATGATATCTAC 411

Tsr-E.coli GACCCG-CGTCAGAGCACCGCCGCAGCGGCAGAGATC--AAACGTAATTACGATATTTAT 396

Aer-E.coli ATCGCGGCGGTGGAGCCGCTGTACAAAGCGTTGAACGCCGGACGTACCAGTAAGCGTATT 426

mrmI-STy -------------------------------------------ATGTTGAGAAATA---- 13

mrmI-STm -------------------------------------------ATGTTGATAAATA---- 13

Tsr-Stm CACGGCGCGTTGGCGGAGCTTATTCAGCTTCTTGGCGCGGGTAAGATTAACGAGTTTTTT 471

Tsr-E.coli CACAATGCGCTGGCGGAGCTGATCCAACTGTTAGGTGCAGGCAAAATCAACGAGTTCTTT 456

*

Aer-E.coli CATAAAGGCCTGGTGGTGCGTAAAGGCTGGCTGGGT--AAACTGCCTTCATTACCGCTTC 484

mrmI-STy -----TCAGCGTCAGGACATGCATTATTCTATTCATGGTATGTACGTTTCTTTTA----- 63

mrmI-STm -----TCAGCGTCAGGACATGCATTATTCTATTCATGGTATGTGCGTTTCTTTTG----- 63

Tsr-Stm GATCAACCGACTCAAAGCTATCAGGACGCTTTTGAGAAGCAGTACATGGCCTAT------ 525

Tsr-E.coli GATCAGCCGACCCAGGGATATCAGGACGGTTTCGAGAAGCAGTATGTGGCTTAC------ 510

* * * * *

Aer-E.coli GCTGGCGGGCGCGTGGAGTGATGACCCTGATGTTTATCTTGCTGGCGGCCATGCTTT--- 541

mrmI-STy ----GTGGATACTTTACAAATAACCTTTCTGCACGATCTCCCTATTTTAATTACGTG--- 116

mrmI-STm ----GTGGATACTTTACAAATAGCTTTTCTGCACGATCTCCCTATTTTAATTACGTG--- 116

Tsr-Stm ----------ATGCAGCAAAACGATCGTCTGTACGATATTGCTGTTGAGGATAACAACAG 575

Tsr-E.coli ----------ATGGAGCAAAACGATCGGCTCCATGATATCGCCGTCAGCGATAACAATGC 560

** * * *

Aer-E.coli --------------------GGTTTGTTGCTGCCCCGGTGGTGACGTATATCCTCTGTGC 581

mrmI-STy ------------------------------------TAATATCATTTATCTGATCTCGGC 140

mrmI-STm ------------------------------------TAATATCATTTATCTGATCTCGGC 140

Tsr-Stm TTCCTACAACCAGGCGATGTGGGTACTGGTCAGTGTGCTGATTGCCGTTCTGGTGGTCAT 635

Tsr-E.coli CTCCTACAGCCAGGCGATGTGGATTCTGGTGGGCGTGATGATCGTCGTACTGGCGGTCAT 620

* *

Aer-E.coli GTTAGTGGTATTGTTGGCA---AGCGCCTGTTTTGAATGGCAGATTGTGCGCCCGATAGA 638

mrmI-STy ATTACTGCTTTGGTGGTATATGACCTGCTATCTGGTAGTGCCAATTAATACCGCGAAGAA 200

mrmI-STm ATTACTGCTTTGGTGGTATATGACCTGCTATCTGGTAGTGCCGATTAATACCGTGAAGAA 200

Tsr-Stm TATCGCCGTCTGGTTCGGCATCAAACTGTCGCTTATCGCCCCGATGAATCGTCTGATTGA 695

Tsr-E.coli CTTCGCCGTCTGGTTCGGTATTAAAGCCTCGCTGGTAGCGCCAATGAATCGCCTGATTGA 680

* * * ** * * * * ** ** *

Aer-E.coli AAATGTTGCCCATCAGGCACTGAAGGTGGCGACCGGAGAACGTAATAGTGTTGAGCATCT 698

mrmI-STy AAGTATTGAAGAGG------------TCGCAGCAGGAAACTTGTCTATTCATATCTCCGA 248

mrmI-STm AAGTATTGAAGAGG------------TCGCAGCAGGAAACTTGTCTATTCATATCTCCGA 248

Tsr-Stm AAGCATTCGTCATA------------TCGCCAGCGGCGATCTGGTGAAGCGTATCGACGT 743

Tsr-E.coli CAGCATTCGTCATA------------TTGCAGGCGGCGATCTGGTGAAACCGATTGAGGT 728

* ** * * ** ** * *

Aer-E.coli GAATCGCAGCGATGAGCTGGGGCTGACATTACGTGCGGTAGGGCAACTTGGCCTGATGTG 758

mrmI-STy ATTTGGCAATAATTGCGCCGGGCGACTCATCCCCGGGATTAATAGCCTGTCGGAGAATAT 308

mrmI-STm ATTTGGCAATAATTGCGCCGGGCGACTCATCCCCGGTATTAATAGCCTGTCGGAGAATAT 308

Tsr-Stm GGAAGGCTCCAACGAAATGGGGCAGTTGGCTG---------AAAACCTGCGTCATATGCA 794

Tsr-E.coli GGATGGCTCTAATGAGATGGGGCAACTGGCAG---------AGAGTTTGCGCCATATGCA 779

** * **** * *

Aer-E.coli CCGTTGGCTAATTAACG---------ATGTCTCAAGCCAGGTGTCCAGTGTCAGAAATGG 809

mrmI-STy TTCCGCGCTGGTACGTGA---------AATCAGATCCTCTTCGCAAACGGCAATGACCCT 359

mrmI-STm TTCCGCGTTGGTACGTGA---------AATCAGATCCTCTTCGCAAACGGCAATGACCCT 359

Tsr-Stm AAGTGAACTGATGCGTACCGTGGGCGATGTACGTAACGGCGCGAATGCGATCTATAGCGG 854

Tsr-E.coli GGGAGAGCTGATGCGTACCGTCGGTGATGTGCGCAACGGGGCCAATGCCATCTATAGCGG 839

* * * * *

Aer-E.coli CAGTGAGACGCTGGCGAAAGGCACCGATGAACTGAACGAACATACCCAGCAGACAGTTGA 869

mrmI-STy TTCCGAACAACTGGCGGCGCGCAGTCTGTCGCTATCCGTAAAGACGGAACAGCAGTCGGC 419

mrmI-STm TTCCGAACAACTGGCGGCGCGCAGTCTGTCGCTATCCGTAAAGACGGAACAGCAGTCGGC 419

Tsr-Stm CGCCAGCGAGATTGCGATGGGCAACAACGATCTCTCTTCCCGTACTGAGCAGCAGGCAGC 914

Tsr-E.coli TGCCAGCGAAATCGCCACCGGCAATAACGATCTCTCTTCGCGCACCGAGCAACAGGCCGC 899

* ** *** ** ** * ** *

Aer-E.coli TAACGTTCAGCAAACGGTGGCGACCATGAACCAAATGGCGGCGTCGGTGAAACAGAACTC 929

mrmI-STy CTCGTTAATTCAAACTGCCGCCAGTATGGATGAAATGGCGGCGAGTACCAAAAACAATGC 479

mrmI-STm CTCGTTAATTCAAACTGCCGCCAGTATAGATGAAATGGCGGCGAGTACCAAAAACAATGC 479

Tsr-Stm GTCTCTGGAAGAGACCGCCGCCAGTATGGAACAACTGACCGCCACCGTGAAACAGAACGC 974

Tsr-E.coli TTCGCTGGAAGAGACGGCAGCCAGCATGGAGCAACTGACCGCAACGGTGAAGCAGAACGC 959

* * ** * ** * ** * ** ** * ** ** * ** *

Aer-E.coli TGCCACGGCGTCGGCTGCCGATAAACTGTCAATCACTGCCAGTAATGCGGCAGTGCAGGG 989

mrmI-STy GGATAATACCCGAATGGCGAGCATACAGGCGGATTGTGCAACCCAATGCGCCCGTAAAGG 539

mrmI-STm GGATAATACCCGAATGGCGAGCATACAGGCGGATTGTGCAACCCAATGCGCCCGTAAAGG 539

Tsr-Stm CGAAAACGCCCGTCAGGCCAGTCACCTGGCGCTGAGTGCGTCAGAGACAGCGCAAAAAGG 1034

Tsr-E.coli CGAAAATGCGCGCCAGGCCAGCCATCTGGCGTTAAGTGCTTCTGAAACGGCGCAACGCGG 1019

* * * ** * * * *** * ** **

Aer-E.coli TGGGGAGGCGATGACCACGGTGATCAAGACAATGGACGATATCGCCGACAGTACCCAGCG 1049

mrmI-STy CGGTGAGTTAATGGTGCGGGTTACAGAAAATATGCGTTCTATTACCGACTGTGCCTCGCA 599

mrmI-STm CGGTGAATTAATGGTGCGGGTTACAGAAAATATGCGTTCTATTACCGACTGTGCCTCGCA 599

Tsr-Stm CGGCAAAGTGGTGGATAACGTCGTACAAACAATGCGCGATATCGCCTCCAGTTCGCAGAA 1094

Tsr-E.coli CGGTAAAGTGGTAGATAACGTGGTGCAGACTATGCGCGATATCTCCACCAGTTCGCAGAA 1079

** * * ** * * *** *** ** * ** * *

Aer-E.coli CATTGGCACCATTACTTCGCTGATTAACGATATTGCGTTTCAGACCAATATTCTGGCCCT 1109

mrmI-STy GATGACGGAGATTATTTCATTGATTGACGGTATCGCATTCCAGACTAATATTCTGGCGCT 659

mrmI-STm GATGACGGAGATTATTTCATTGATTGACGGTATCGCATTCCAGACTAATATTCTGGCGCT 659

Tsr-Stm AATCGCCGATATTATCAGCGTAATCGACGGTATTGCTTTCCAGACCAATATTCTGGCGCT 1154

Tsr-E.coli AATCGCCGATATTATCAGCGTAATTGACGGCATTGCCTTCCAGACCAATATTCTGGCTTT 1139

** **** * ** *** ** ** ** ***** *********** *

Aer-E.coli GAATGCGGCGGTGGAAGCGGCGCGTGCCGGCGAACAGGGCAAAGGTTTTGCAGTGGTGGC 1169

mrmI-STy TAACGCGGCGGTAGAGGCTGCGCGGGCGGGCGATCATGGAAAAGGTTTCTCCGTGGTGGC 719

mrmI-STm TAACGCGGCGGTAGAGGCTGCACGGGCGGGCGATCATGGAAAAGGTTTCTCCGTGGTGGC 719

Tsr-Stm GAATGCGGCGGTAGAAGCGGCGCGCGCAGGCGAGCAGGGACGCGGGTTCGCAGTGGTGGC 1214

Tsr-E.coli GAACGCGGCGGTTGAGGCTGCGCGTGCGGGTGAGCAAGGGCGCGGTTTTGCGGTGGTCGC 1199

** ******** ** ** ** ** ** ** ** ** ** ** ** * ***** **

Aer-E.coli AGGGGAAGTGCGTCATTTAGCCAGCCGCAGCGCTAATGCTGCCAACGATATTCGCAAGCT 1229

mrmI-STy CAGGGAAGTGCGTAATCTGGCGCATCGTAGCGCTGAGGCGGCAAAAAATATCAAGGCGCT 779

mrmI-STm CGGGGAAGTGCGTAATCTGGCGCATCGTAGCGCCGAGGCGGCAAAAAGTATCAAGGCGCT 779

Tsr-Stm CGGTGAAGTCCGTAATCTGGCCCAGCGTAGCGCGCAGGCGGCACGGGAGATCAAGAGTCT 1274

Tsr-E.coli GGGAGAAGTGCGTAATCTGGCCCAGCGCAGCGCCCAGGCGGCTCGTGAAATTAAAAGCCT 1259

* ***** *** ** * ** ** ***** * ** ** ** **

Aer-E.coli GATTGATGCCAGTGCTGATAAGGTGCAATCCGGTTCGCAGCAGGTACACGCCGCCGGACG 1289

mrmI-STy GATCGACGTTACGCATGACAATGTGCGGCAGGGGGCCGCCATAGTGCAGGAGGCTGAAAA 839

mrmI-STm GATCGACGTTACGCATGACAATGTGCGGCAGGGGGCCGCCATAGTGCAGGAGGCTGAAAA 839

Tsr-Stm GATTGAGGATTCCGTGAGCCGTGTTGATGTAGGTTCGACGCTGGTCGAAAGCGCCGGTGA 1334

Tsr-E.coli GATTGAAGACTCGGTGGGGAAAGTGGATGTTGGCTCTACGCTGGTCGAAAGCGCCGGGGA 1319

*** ** * ** ** * ** * ** *

Aer-E.coli GACGATGGAAGATATTGTGGCACAGGTGAAAAACGTCACCCAGTTGATCGCCCAGATTAG 1349

mrmI-STy AAATATGCAGGAGATTGTTGGCGGCTCCGGGCAATTAAACGTGCTGATGAGTGAAATTTC 899

mrmI-STm AAATATGCAGGAGATTGTTGGCGGCTCCGGGCAATTAAACGTGCTGATGAGTGAAATTTC 899

Tsr-Stm AACCATGGATGAGATCGTCAATGCAGTGACCCGCGTGACCGATATCATGGGCGAGATTGC 1394

Tsr-E.coli AACAATGGCGGAGATTGTCAGCGCCGTGACCCGCGTGACGGACATTATGGGCGAAATTGC 1379

* *** ** ** ** * * * ** * ***

Aer-E.coli CCATTCAACGCTGGAACAGGCCGATGGGCTTTCCAGCCTGACCCGTGCAGTGGATGAGCT 1409

mrmI-STy CACCACCACGCGGGAGCAGGAAAAAGGCATTAACCAGATAACCCTGGCGTTGAGCGATCT 959

mrmI-STm CACCACCACGCGGGAGCAGGAAAAAGGCATTAACCAGATAACCCTGGCGTTGAGCGATCT 959

Tsr-Stm CTCGGCGTCTGACGAGCAAAGCCGTGGTATCGACCAGGTGGGCCTGGCGGTAGCGGAGAT 1454

Tsr-E.coli TTCTGCTTCTGATGAGCAGAGCCGTGGTATCGATCAGGTTGGCTTAGCGGTTGCTGAGAT 1439

* * ** ** ** * * * ** * ** *

Aer-E.coli TAACCTGATCACCCAGAAAAATGCCGAGCTGGTGGAAGAGAGTGCGCAGGTGTCGGCGAT 1469

mrmI-STy GGAAAGCGCAACCCATAGCAATGTCTTAATGGTTGAAGCGCTATCTGCTTCTTCGGATGT 1019

mrmI-STm GGAAAGCGCAACCCATAGCAATGTCTTAATGGTTGAAGCGCTATCTGCTTCTTCGGATGT 1019

Tsr-Stm GGATCGCGTAACGCAGCAGAACGCCTCGCTGGTGGAAGAGTCCGCCGCCGCGGCTGCGGC 1514

Tsr-E.coli GGACCGGGTAACTCAACAGAACGCCGCGCTGGTGGAAGAGTCTGCCGCTGCCGCCGCCGC 1499

* ** ** ** * * **** **** * * * *

Aer-E.coli GGTGAAACACCGCGCCAGCCGACTGGAAGACGCGGTGACGGTACTGCATTAA-------- 1521

mrmI-STy TTTAAAGGCGCAGGTGATCGAGTTACAGACTAAAACCGACAAATTTCGTTTAAGCCAGCC 1079

mrmI-STm TTTAAAGGCGCAGGTGATCGAGTTACAGACTAAAACCGACAAATTTCGTTTAAGCCAGCC 1079

Tsr-Stm GCTGGAAGAGCAAGCCAGCCGTCTGACCCAGGCCGTCGCGGTGTTCCGTATTCACCAGCA 1574

Tsr-E.coli GCTGGAAGAGCAGGCCAGTCGCCTGACCGAAGCAGTGGCAGTGTTCCGGATTCAGCAACA 1559

* * * * * * * *

Aer-E.coli ------------------------------------------------------------ 1521

mrmI-STy GGGTTACAGTGAACATGCGCTGTCGCGCTCTCATG-----TATCACCTCTTT-------- 1126

mrmI-STm GGGTTACAGTGAACATGCGCTGTCGCACTCTCATG-----TATCATCTCTTT-------- 1126

Tsr-Stm ACAGCAGCG--TGCGCGTGAAGTGGCTGCGGTAAAAACCCCGGCAGCCGTGTCGTCACCA 1632

Tsr-E.coli GCAGCGTGA--AACATCGGCTGT------GGTAAAAACCGTGACGCCAGCTGCGCCGCGT 1611

Aer-E.coli --------------------------------------------- 1521

mrmI-STy ----CGACCATCACCAGGCGCGGCCAGGCCTGA------------ 1155

mrmI-STm ----CGACCATCACCAGGCGCGGCCAGGCCTGA------------ 1155

Tsr-Stm AAGGCCGCAGTGGCCGACGGCAGCGATAATTGGGAAACATTTTAA 1677

Tsr-E.coli AAAATGGCCGTGGCAGATAGCGAGGAGAACTGGGAAACATTTTAA 1656

**Supplementary Figure 4**. Clustal analysis of (A) protein sequences and (B) DNA-coding sequences for *Aer_E_*_.coli_, *mrmI*_STy_, *mrmI*_STm_, *Tsr*_STm_, *Tsr*_E.coli_ (*cheD*). Coverage, identity and similarity are summarized in Table 3. Note that the well-known CheD protein and its coding gene are homologous of *Tsr* from *Salmonella*.


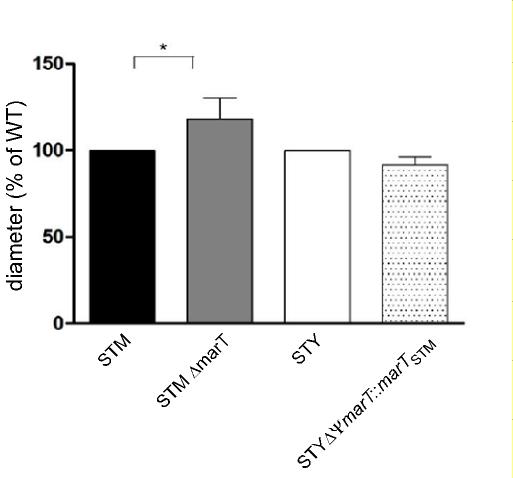
Supplementary Figure 5

**Supplementary Figure 5**. *marT* participates in motility of STM. Bacterial motility was measured by halo growth diameters on semi-solid agar. The plot shows percent growth compared to WT strains, based on 3 independent biological replicates. **p*< 0.05.

Supplementary Figure 6


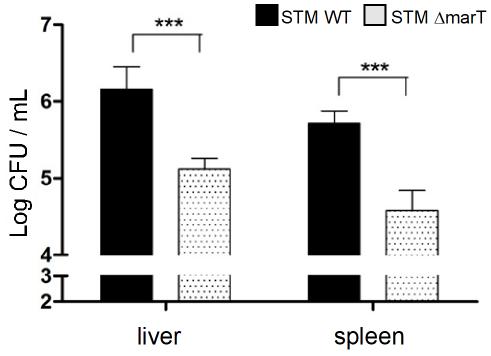


**Supplementary Figure 6.** Deletion of *marT* reduces virulence of *S.* Typhimurium in orally infected mice. The CFU/mL of STM and STM Δ*marT* collected from the liver and spleen of orally infected mice, was calculated. Note that although MarT is a negative modulator of *mrmI* and therefore deletion of *marT* produces increased expression of *mrmI*, the invasion of *Salmonella* is reduced. This observation may be due to direct or indirect regulation of MarT on target genes other than *mrmI*. Representative assay of 3 independent biological replicates is shown. *** *p* < 0.005.

Supplementary Figure 7


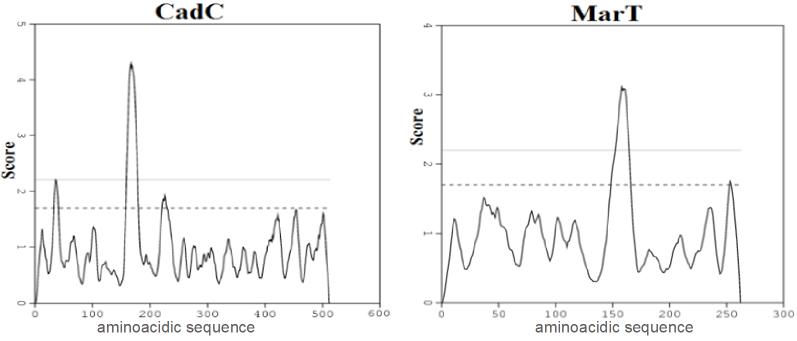


**Supplementary Figure 7.** Prediction of transmembrane domains in CadC and MarT. The presence of transmembrane domains (TMD) was predicted using the DAS (Dense Alignment Surface) suite. In the analysis of proteins CadC and MarT, the continuous horizontal line indicates the most stringent prediction of TMD prediction, while the dotted horizontal line is less stringent. Both CadC and MarT present a single predicted TMD, close to amino acid 160.

## Supplementary Tables

| **Supplementary Table 1**. Used DNA primers | |
| --- | --- |
| **primer use/name** | **sequence 5'-3'** |
| **mutagenesis** |  |
| Wanner *mrmI* Fw | ATGTTGAGAAATATCAGCGTCAGGACATGCATTATTCTATTGTAGGCTGGAGCTGCTTCG |
| Wanner *mrmI* Rv | TCAGGCCTGGCCGCGCCTGGTGATGGTCGAAAGAGGTGATCATATGAATATCCTCCTTAG |
| H1 mrmI-tetR | TAACGCTGTCCACAGTAATAACACGGAGAAACAGTCATTCGGAAAAAGGTTATGCTGCTT |
| H2 mrmI-tetA | TTCTCAACATTTTTGACGGCCTACAACCAGGATAGGAGACATCATTTGGTGACGAAATAA |
| Wanner tetA Fw | ATGAATAGTTCGACAAAGATCGCATTGGTAATTACGTTACTGTAGGCTGGAGCTGCTTCG |
| Wanner tetA Rv | CTAAGCACTTGTCTCCTGTTTACTCCCCTGAGCTTGAGGGCATATGAATATCCTCCTTAG |
| **qPCR** |  |
| *mgtB* qPCR Fwd | GCACCTTAACCCAGGACAATA |
| *mgtB* qPCR Rev | CGCTCTGGCTGCTACTATTC |
| *narQ* qPCR Fwd | CCAACTGGCTGGAGATGAATAG |
| *narQ* qPCR Rev | CGCCAGAACGAAGAGATCAA |
| *mrmI* qPCR Fwd* | CTTTCTGCACGATCTCCCTATT |
| *mrmI* qPCR Rev* | TGCTGCGACCTCTTCAATAC |
| *acrR* qPCR Fwd | TGGAGACACGACAACACATC |
| *acrR* qPCR Rev | AGCAGCGTTCGCAATCT |
| marT 1 qPCR Fwd | GTGTCGGCAAATACGCTTTATC |
| marT 1 qPCR Rev | CTACGCGGAACCGTGATTAT |
| 16S qPCR FW** | TACCTGGTCTTGACATCCAC |
| 16S qPCR RV** | TTATCACTGGCAGTCTCCTT |
| The qPCR primers possess 100% identity to intragenic regions in their respective target gene. *Used for genotypic confirmation. ** Used for both STM and STY. | |
